# Neuromuscular fatigue and recovery after strenuous exercise depends on skeletal muscle size and stem cell characteristics

**DOI:** 10.1038/s41598-021-87195-x

**Published:** 2021-04-08

**Authors:** Philipp Baumert, S. Temple, J. M. Stanley, M. Cocks, J. A. Strauss, S. O. Shepherd, B. Drust, M. J. Lake, C. E. Stewart, R. M. Erskine

**Affiliations:** 1 Exercise Biology Group, Faculty of Sport and Health Sciences, Technical University of Munich, Munich, Germany; 2 Research Institute for Sport & Exercise Sciences, Liverpool John Moores University, Liverpool, UK; 3 School of Sport, Exercise and Rehabilitation Sciences, College of Life and Environmental Sciences, University of Birmingham, Birmingham, UK; 4 Institute of Sport, Exercise & Health, University College London, London, UK

**Keywords:** Physiology, Neurophysiology

## Abstract

Hamstring muscle injury is highly prevalent in sports involving repeated maximal sprinting. Although neuromuscular fatigue is thought to be a risk factor, the mechanisms underlying the fatigue response to repeated maximal sprints are unclear. Here, we show that repeated maximal sprints induce neuromuscular fatigue accompanied with a prolonged strength loss in hamstring muscles. The immediate hamstring strength loss was linked to both central and peripheral fatigue, while prolonged strength loss was associated with indicators of muscle damage. The kinematic changes immediately after sprinting likely protected fatigued hamstrings from excess elongation stress, while larger hamstring muscle physiological cross-sectional area and lower *myoblast:fibroblast* ratio appeared to protect against fatigue/damage and improve muscle recovery within the first 48 h after sprinting. We have therefore identified novel mechanisms that likely regulate the fatigue/damage response and initial recovery following repeated maximal sprinting in humans.

## Introduction

Hamstring strain is the most frequently occurring injury in sport^1^, particularly in those sports that involve high-speed running^2^. Although the aetiology is unclear, numerous risk factors have been proposed, such as short fascicle length, poor flexibility, poor hamstring strength, and inadequate warm-up^3^. Further, it is unknown whether hamstring strain is the result of a single event that exceeds the physiological range of hamstring muscle extensibility and contractility, or as a result of an accumulation of eccentric contractions during repeated maximal sprints, causing neuromuscular fatigue^3^. Neuromuscular fatigue is responsible for acute, as well as prolonged, impairment of muscle function, classified as central fatigue (i.e. originating in the central nervous system), or peripheral fatigue (i.e. distal to the neuromuscular junction)^4^. Although it was recently reported that both central and peripheral fatigue contribute to impaired hamstring muscle function immediately after repeated maximal sprint-related interventions^5,6^, the contribution of neuromuscular fatigue to hamstring muscle impairment and recovery, following repeated maximal sprints over time, is insufficiently studied^7^. An understanding of hamstring neuromuscular fatigue following repeated maximal sprints may be crucial for understanding hamstring strain aetiology.

Peripheral fatigue may be caused by ultrastructural muscle damage, which is indicated by Z-line disturbance^8^ as well as disruption of the extracellular matrix^9^. The extracellular matrix comprises different layers of connective tissue and surrounds the muscle fibres, fascicles and the entire muscle^10^. It provides structural scaffolding for muscle remodelling and plays an integral role in force transmission, in particular the fascicle surrounding structure known as the perimysium^11^. This is referred to as exercise-induced muscle damage and it is exhibited by prolonged strength loss and delayed-onset muscle soreness, as well as the release of muscle-specific proteins [e.g. creatine kinase (CK)] into the circulation over the following days^12^. After substantial muscle damage, myogenic satellite cells (skeletal muscle stem cells), play a key role in skeletal muscle regeneration and remodelling^13^. Activated satellite cells (myoblasts) proliferate and migrate from their niche along the basal lamina to the injury site before terminally differentiating and fusing with damaged myofibrils to repair injury. There is increasing evidence that fibroblasts, the main cell type of muscle connective tissue, also play a critical role in supporting muscle regeneration^14,15^. Following damage, infiltrating inflammatory cells activate muscle fibroblasts, which proliferate and migrate to the area of the myotrauma and produce extracellular matrix components in an orchestrated and regulated fashion to support healthy muscle remodelling^15,16^. The finely-tuned, coordinated resolution and restructuring of the ECM is crucial for healthy muscle remodelling^17,18^. Interaction of activated satellite cells with fibroblasts helps to dissolve and reorganise the ECM by suppressing the master regulator of collagen biosynthesis Rrbp1 in the days and weeks after the injury to avoid long lasting unfavourable fibrosis and to support healthy muscle regeneration^19,20^. There is an increasing number of investigations into the effect of fibroblasts on skeletal muscle regeneration following injury, including models such as electrical stimulation or barium chloride^14,15^. However, little is known about the role of fibroblasts during the initial response and recovery following *physiological* exercise-induced muscle damage, e.g. following repeated sprinting.

Acute damage to the muscle–tendon complex may facilitate hamstring strain, which is thought to occur in the late swing phase of sprinting, when the hamstring muscles contract eccentrically, i.e. trying to shorten while being forcibly lengthened in an attempt to decelerate the shaft before initial foot–ground contact^21^. Therefore, a short biceps femoris long head (BF_LH_) fascicle length has been suggested to increase hamstring strain risk^22^, as the BF_LH_ is thought to be relatively more eccentrically stretched during the late swing phase of sprinting compared to the other hamstring muscles^21^. However, no study has investigated the relationship between BF_LH_ architecture (including muscle fascicle length and cross-sectional area), and the prolonged hamstring muscle response to exercise-induced neuromuscular fatigue. Finally, lower limb neuromuscular fatigue might cause a number of biomechanical alterations in running kinematics^23^. However, it is not known whether repeated maximal sprints influence kinematic patterns, and whether this can lead to prolonged changes in lower-limb kinematics, which may play a role in the development of muscle strain following insufficient recovery^3^.

Here we demonstrated that both central and peripheral fatigue, assessed via surface electromyography (sEMG) and electrical stimulation, contributed to the immediate loss of muscle function in both the quadriceps and hamstring muscle groups, but that peripheral factors mainly contributed to the sustained loss of hamstring muscle function. Moreover, we established that a lower myoblast:fibroblast ratio in isolated primary human muscle stem cells correlated with improved recovery from both repeated maximal sprints and an in vitro artificial wounding assay within the first 48 h. We also report that BF_LH_ architecture (i.e. physiological cross-sectional area, PCSA) was associated with hamstring fatigue, and that neuromuscular fatigue led to reduced knee extension during the late swing phase of steady-state running. Thus, with this interdisciplinary study, we have identified novel cellular and neuromuscular mechanisms underpinning central and peripheral fatigue following repeated sprinting, which ultimately led to kinematic changes during the running stride phase associated with hamstring strain injury*.*

## Results

### Effect of the repeated maximal sprint intervention on neuromuscular fatigue

**Figure 1:**
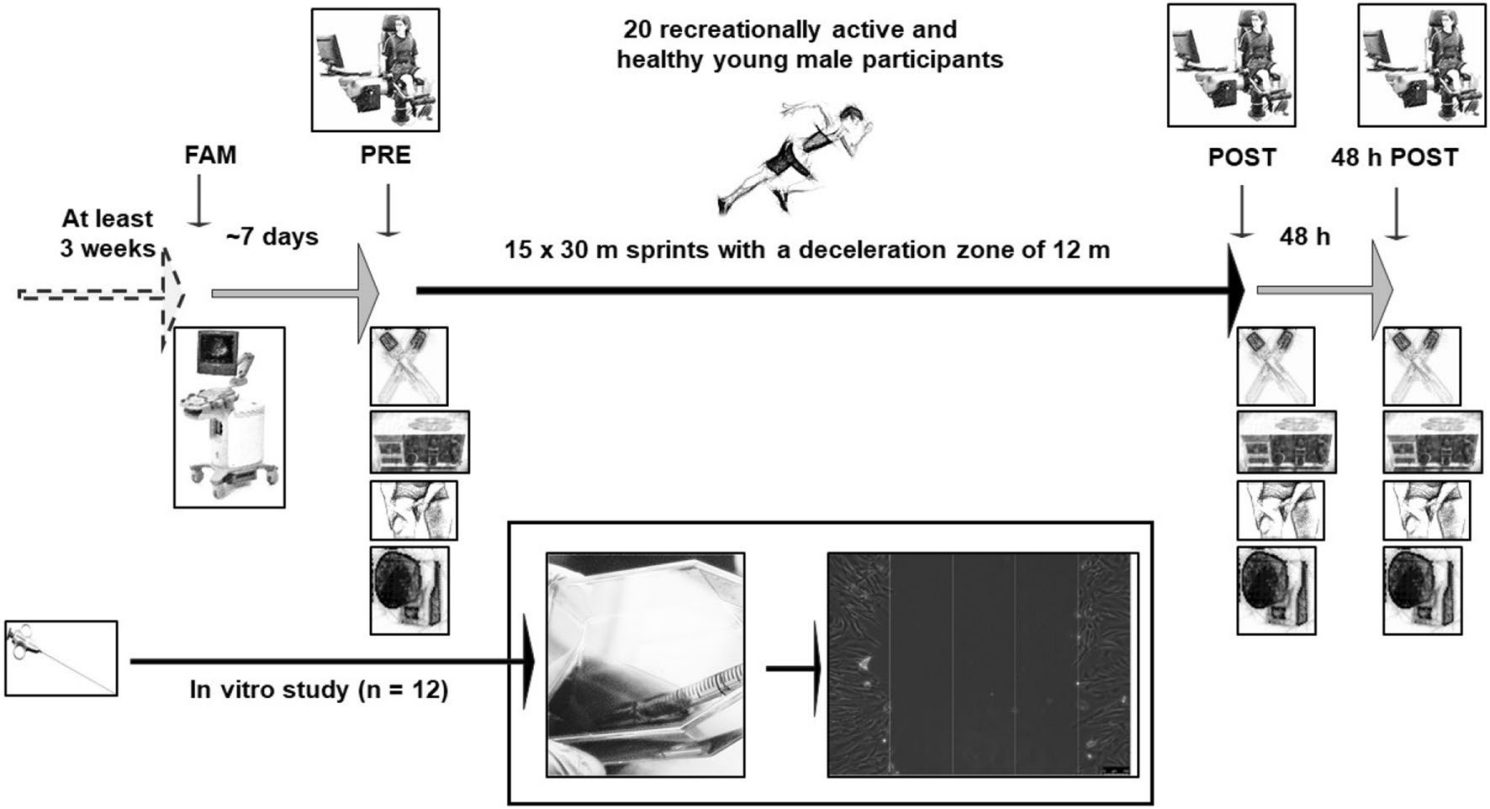
Study design of the repeated maximal sprint intervention. *FAM* familiarisation, *PRE* before the repeated maximal sprint intervention, *POST* after the repeated maximal sprint intervention.

We investigated the effect of neuromuscular fatigue/damage following a repeated maximal sprint intervention that comprised 15 × 30 m sprints with a deceleration zone of 12 m (Fig. 1). Each sprint was separated by a rest of 90 s, and after every 5th repetition, the participants were allowed to rest for 3 min. To examine the effect of repeated maximal sprints on neuromuscular fatigue, we measured different fatigue parameters before (PRE), immediately after (POST), and 48 h after (POST48) the repeated maximal sprint intervention. The average 30 m sprinting speed was 6.48 ± 0.33 m s^−1^. There was a main effect of time for heart rate, 30 m sprinting time, rating of perceived exertion and lactate concentration, with all parameters increasing from PRE to POST (all P < 0.001). Blood lactate concentration increased from PRE (1.63 ± 0.45 mmol/L) to POST (9.82 ± 3.62 mmol/L; P < 0.001). The sprinting performance (measured via the performance decrement score^24^) decreased by 3.98 ± 2.99% during the run, and rating of perceived exertion increased by 96.5 ± 35.2% from PRE-to-POST, indicating fatigue had occurred.

We then performed in vivo functional analysis to assess if repeated maximal sprints resulted in an increase in central and/or peripheral fatigue. We, therefore, measured BF_LH_ muscle activation via normalised surface electromyography (sEMG) during hamstring maximum voluntary contraction (MVC). We observed a change in sEMG (F_F2,24_ = 4.35, P = 0.022), with post-hoc pairwise comparisons revealing a decrease from PRE-to-POST (-24.3%; P = 0.019). However, this change was no longer evident at POST48 (P = 0.157, Table 1), suggesting that central fatigue occurred immediately after repeated maximal sprints. No other changes in muscle (co)activation were observed at any time point (P > 0.05).

**Table 1:** Effect of the repeated maximal sprint intervention on measures of muscle activation.

Assessment (unit)	n	PRE	POST	POST48	F-test	P value
Hamstring muscle voluntary activation (ITT, %)	20	98.5 ± 2.64	94.1 ± 7.83	96.9 ± 5.96	F (1.4,25.8) = 2.75	0.099
Normalised BF_LH_ knee flexion sEMG_max_ (%)	14	3.32 ± 1.33	2.27 ± 0.72*	2.85 ± 1.16	F (2,24) = 4.35	0.022
Vastus lateralis knee extension sEMG_max_ (mV)	16	0.49 ± 0.29	0.46 ± 0.33	0.50 ± 0.32	F (2,30) = 0.22	0.726
Quadriceps CoA during 30° hamstring MVC (%)	13	5.41 ± 7.02	4.02 ± 6.57	4.20 ± 8.40	F (2,24) = 0.42	0.663
Hamstring CoA during 80° quadriceps MVC (%)	11	4.71 ± 3.21	6.12 ± 3.76	7.29 ± 4.01	F (2,20) = 1.66	0.216

Data are presented as mean ± SD. One-way ANOVA, F- and P-values are reported.

*ITT* interpolated twitch technique, *BF*_*LH*_ biceps femoris long head, *CoA* co-activation, *sEMG* surface electromyography.

*Different to PRE (P < 0.05).

**Figure 2:**
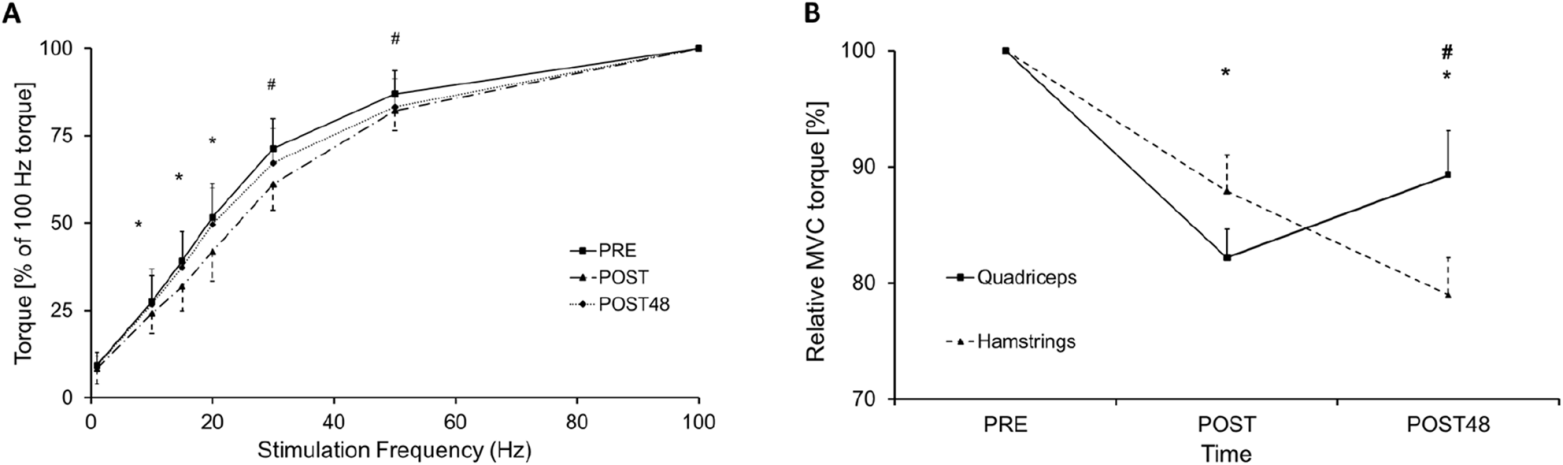
(**A**) Torque-frequency relationship, all frequencies were normalised to 100 Hz. *Significant differences between before (PRE) and immediadtely after (POST) the repeated maximal sprint intervention, P < 0.05; ^#^significant differences between PRE and POST, and between PRE and POST48, P < 0.05. Data are presented as mean ± SEM. (**B**) Comparison of relative maximal voluntary contraction (MVC) loss between hamstring and quadriceps muscle group before (PRE), immediately after (POST) and 48 h after (POST48) the repeated maximal sprint intervention. *Signifcant differences compared to PRE, P < 0.001; ^#^significant differences between quadriceps and hamstring MVC, P < 0.05. Data are expressed as mean ± SEM. This figure was produced in Microsoft Excel 2016.

We also assessed the torque-frequency relationship in vivo via electrical stimulation to indicate peripheral (muscle) fatigue. There was an interaction between time × stimulation frequency (n = 19; F_4.9,88.2_ = 6.62, P < 0.001; Fig. 2). Post-hoc paired t-tests revealed differences PRE-to-POST for 10–50 Hz (P < 0.05), but lower frequencies between 10 and 20 Hz reverted to baseline values POST48 (P > 0.05), while the frequencies of 30 and 50 Hz were still decreased POST48 compared to their baseline values (P < 0.05), providing evidence that peripheral fatigue occurred immediately after the repeated sprints and remained for 48 h.

### Effect of the repeated maximal sprint intervention on MVC strength, muscle soreness and serum markers of exercise-induced muscle damage

To investigate the effect of repeated maximal sprints on biomarkers of exercise-induced muscle damage, we assessed hamstring (knee flexion) and quadriceps (knee extension) MVC, muscle soreness, serum creatine kinase (CK) activity and interleukin-6 (IL-6) concentrations PRE, POST and POST48. Isometric hamstring and quadriceps MVC, muscle soreness (all P < 0.001) and serum CK activity (F_1.3,21.8=_5.98, P = 0.017), as well as IL-6 concentration (F_1.3,21=_5.96, P = 0.018), showed a main effect of time, which are indicators of muscle damage (Table 2) that was similar to other studies^7,25,26^. Post-hoc pairwise comparisons revealed that, compared to PRE, both serum CK activity (+ 93.0%) and serum IL-6 concentration (+ 307%) were elevated at POST (both P = 0.027), and CK activity further increased at POST48 (+ 256%; P = 0.012), while serum IL-6 concentration reverted to baseline values (P > 0.05).

**Table 2:** Effect of the repeated maximal sprint intervention on muscle damage-biomarkers.

Assessment (unit)	PRE	POST	POST48	F-test	P value
Quadriceps MVC (N⋅m)	270.5 ± 51.6*	222.4 ± 52.5*	243.0 ± 71.3*	F(2,38) = 16.55	< 0.001
Hamstring MVC (N⋅m)	142.5 ± 25.0*	124.8 ± 29.9*	112.4 ± 30.1*	F(2,38) = 25.12	< 0.001
Squat Muscle soreness (cm)	0.20 ± 0.41*	1.95 ± 1.61*	2.87 ± 1.71*	F(2,38) = 28.62	< 0.001
Lunge Muscle soreness (cm)	0.30 ± 0.57	2.30 ± 2.08^†^	3.48 ± 2.07^†^	F(2,38) = 17.02	< 0.001
Range of Motion (°)	120.3 ± 6.76	115.7 ± 6.77^†^	116.0 ± 6.27†	F(2,38) = 9.33	< 0.001
CK activity (mU/mL)	27.9 ± 23.3	53.8 ± 45.3^†^	99.3 ± 104.5^†^	F(1.3,21.8) = 5.98	0.017
IL-6 concentration (pg/mL)	1.89 ± 3.10	7.68 ± 9.95^#^	1.59 ± 3.46	F(1.3,21) = 5.96	0.018

Values are mean ± SD. One-way ANOVA, F- and P-values are reported.

*MVC* maximal voluntary contraction, *CK* creatine kinase, *IL-6* interleukin-6.

*Significant differences between all time points.

^†^Differences PRE-to-POST and PRE-to-POST48 (P < 0.05).

^#^Differences PRE-to-POST and POST-to-POST48 (P < 0.05).

Further, there was an interaction between time and muscle groups concerning relative MVC torque loss (percentage change from PRE MVC) (F_2,76_ = 7.23, P = 0.001). Relative MVC decreased similarly in both quadriceps and hamstring muscle groups PRE-to-POST (Fig. 2). However, at POST48, hamstring MVC continued to decrease from POST (− 9.26%; P = 0.010), while quadriceps MVC began to return to PRE values (+ 8.47%; P = 0.016) and was higher than hamstring MVC at POST48 (P = 0.034).

### Effect of the repeated maximal sprint intervention on lower-limb kinematics

To assess the consequential effect of neuromuscular fatigue on lower-limb kinematics, we captured treadmill running (4.17 m s^−1^) kinematics with an eight-camera motion capture system over time. Three-dimensional motion analysis demonstrated that, despite significance not being achieved, there was a tendency towards a longer running cycle time POST (+ 1.01%) and POST48 (+ 1.86%), compared to PRE (P = 0.080; Table 3). Further, treadmill running demonstrated decreased peak knee extension (P = 0.047) during the late swing phase at POST (− 10.9%) compared to PRE, but this reverted to baseline POST48. The percentage change in peak knee extension correlated with the percentage change in relative hamstring MVC torque both measured POST-to-POST48 (R^2^ = 0.26, F_1,2_ = 5.673, P = 0.031).

**Table 3:** Effect of the repeated maximal sprint intervention on kinematics of treadmill running at 4.17 m s^−1^.

Kinematics (unit)	PRE	POST	POST48	F-test	P value
Peak knee flexion (swing phase) (°)	− 103 ± 13.9	− 110 ± 12.4	− 108 ± 12.6	F(2,20) = 2.84	0.082
Peak knee extension (swing phase) (°)	− 3.66 ± 5.32	− 7.08 ± 5.07*	− 4.29 ± 7.06	F(2,20) = 3.57	0.047
Contact hip flexion (toe strike) (°)	26.3 ± 4.06	29.2 ± 8.21	23.0 ± 11.2	F(2,16) = 1.30	0.299
Contact knee flexion (toe strike) (°)	− 15.0 ± 6.25	− 17.6 ± 6.79	− 13.3 ± 9.64	F(2,22) = 2.79	0.083
Duration Running cycle (s)	0.67 ± 0.03	0.68 ± 0.03	0.69 ± 0.02	F(2,20) = 2.88	0.080
Stance phase duration (s)	0.18 ± 0.02	0.19 ± 0.02	0.19 ± 0.03	F(1.3,13.1) = 1.01	0.356
Swing Phase (s)	0.50 ± 0.04	0.50 ± 0.05	0.50 ± 0.03	F(1.2,12) = 0.03	0.899

Values are mean ± SD. One-way ANOVA, F- and P-values are reported.

Knee fully extended = 0°; negative value indicates a flexed knee.

*Different to PRE (P < 0.05).

### Architecture of the biceps femoris long head muscle

**Figure 3:**
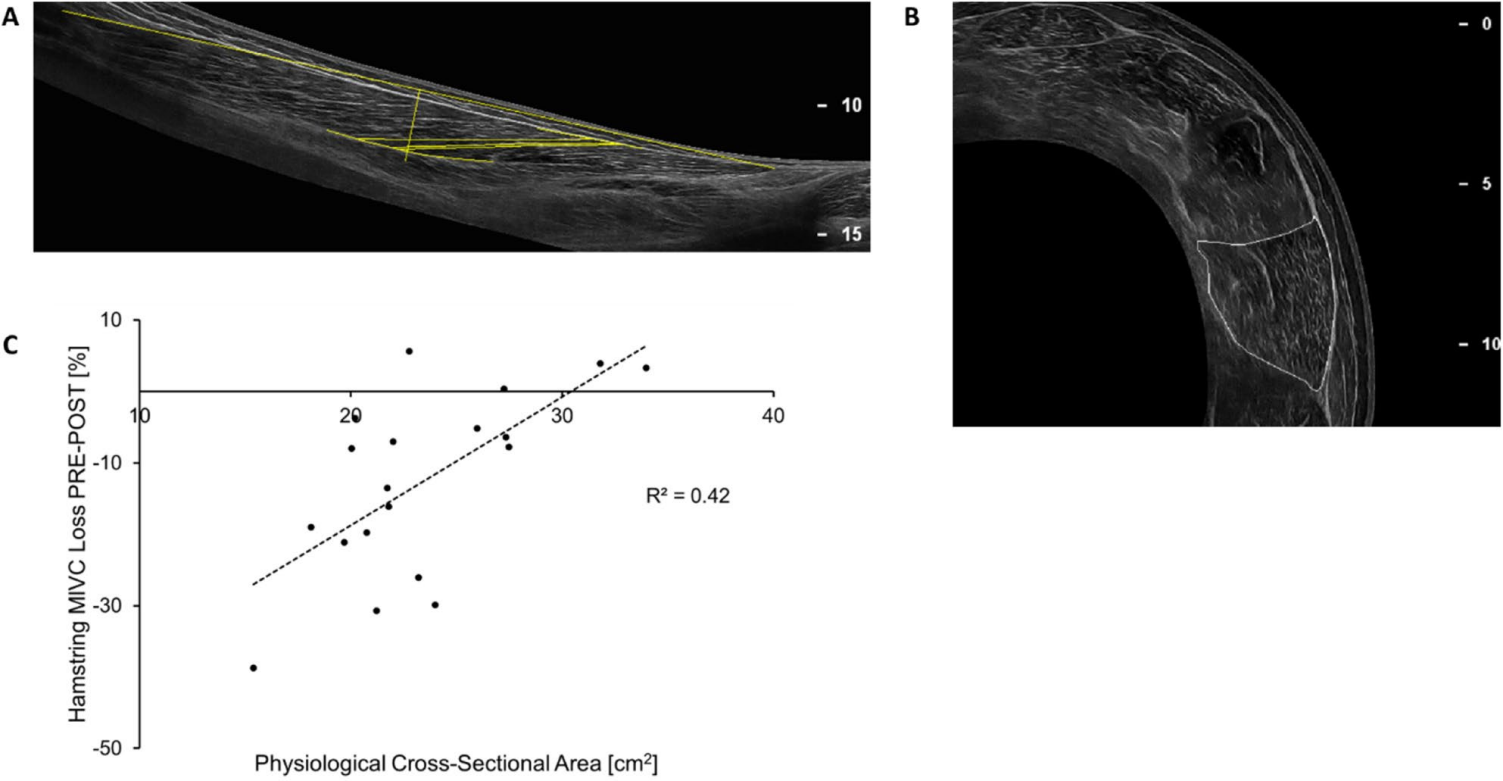
(**A**) Longitudinal image of biceps femoris long head, assessment of the biceps femoris long head is highlighted (total muscle length and fascicle length together with pennation angle at 50% of total muscle length). (**B**) Cross-sectional image at 60% muscle length (= 100% proximal myotendinous junction), biceps femoris long head is highlighted. (**C**) Correlation between biceps femoris long head muscle physiological cross-sectional area and % hamstring maximum isometric voluntary contraction (MIVC) decrease from before (PRE) to immediately after (POST) the repeated maximal sprint intervention (P = 0.003). This figure (**C**) was produced in Microsoft Excel 2016.

To assess whether architectural parameters of the BF_LH_ muscle (Fig. 3) were associated with markers of peripheral fatigue, we performed ultrasound measurements of the BF_LH_ muscle (Table supplement 4). Muscle fascicle length and pennation angle of the BF_LH_, which have previously been linked to hamstring muscle strain risk^27^, did not correlate with any outcome variable of neuromuscular fatigue. However, BF_LH_ PCSA (mean ± SD: 23.4 ± 4.62 cm^2^) correlated inversely with relative hamstring MVC loss PRE-to-POST (R^2^ = 0.42, F_1,17_ = 12.37, P = 0.003, Fig. 3).

### Artificial wound healing assay to investigate repair and regeneration regarding myoblast:fibroblast ratio

Preliminary data from our laboratory demonstrated that skeletal muscle stem cell composition (i.e. myoblast:fibroblast ratio), derived from two volunteers with ratios representing the extreme conditions of the myoblast:fibroblast percentage, played a role in the success of artificial wound healing in vitro^28^. Human primary skeletal muscle stem cells with a high myoblast:fibroblast ratio resulted in reduced cell migration into an artificial wound (assessed by cell number within the wound) when compared with stem cells with a low myoblast:fibroblast ratio. No significant differences were observed in the relative proportion of migrating cells (i.e. between myoblasts and fibroblasts) over 48 h^29^. Further, recent investigations have revealed that the skeletal muscle stem cell ratio does not change during in vitro cell culturing^30,31^. We, therefore, further assessed the effect of the myoblast:fibroblast ratio on skeletal muscle recovery following in vitro artificial wounding to extend the preliminary in vitro results from our laboratory. To be able to demonstrate a coefficient of determination of ≥ 0.50 between myoblast:fibroblast ratio and our dependent variables, a priori power calculations for a Pearson's r correlation was run using G*Power (version 3.1.9.1), and it revealed that 11 persons provided alpha = 5%, and power = 80%. Thus, we used primary human skeletal muscle stem cells derived from 12 participants, six who participated in both the repeated maximal sprint intervention and also volunteered to provide a muscle biopsy at least three weeks before the repeated maximal sprint intervention, and another six (two male and four females), who did not participate in the repeated maximal sprint intervention (to increase the power of the in vitro study). The mononuclear cells, which included muscle stem cells, were isolated, cultured and then characterized by immunofluorescence staining. The mean ± SD myoblast:fibroblast ratio of the twelve participants was 1.26 ± 1.00 (range 0.276–2.93). We did not detect any differences in muscle stem cell characteristics between cells obtained from females and males (data not shown). We, therefore, combined the data from all muscle cells and correlated the muscle characteristics with individual myoblast:fibroblast ratios. We observed no correlations regarding myoblast:fibroblast ratio and the total number of skeletal muscle stem cells (combined number of myoblasts, fibroblasts and other stem cells) migrating into the artificial wound within all three segments combined at 24 h (R^2^ = 0.20, F_1,10_ = 2.56, P = 0.141) or 48 h (R^2^ = 0.02, F_1,10_ = 0.19, P = 0.671) after the scratch assay.

**Figure 4:**
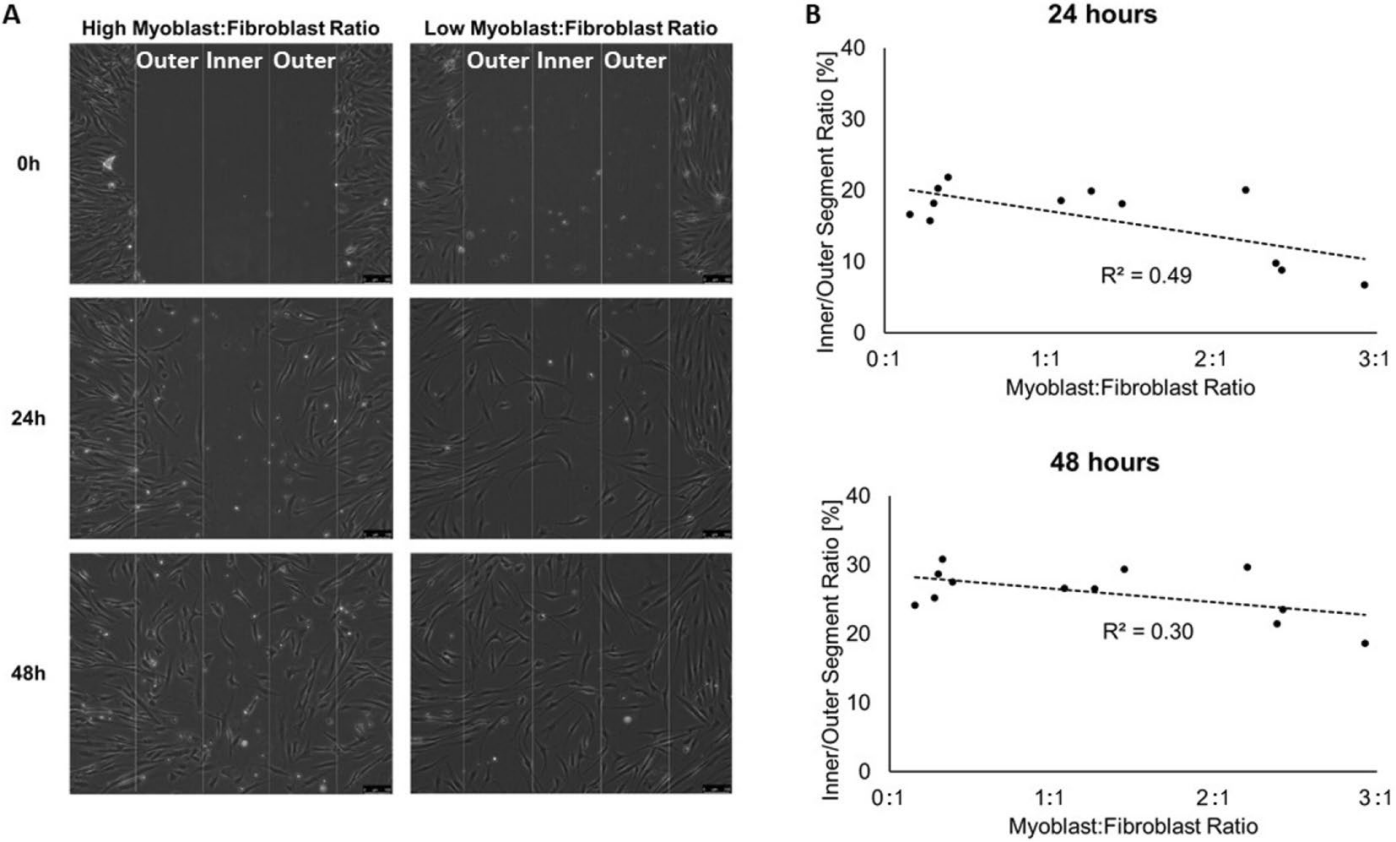
(**A**) Representative images for cell migration of muscle cells with a high myoblast:fibroblast ratio (2.4; left) and with a low myoblasts:fibroblast ratio (0.3; right) into the artificial wound. The wound area is about 900 µm in width and split into 3 × 300 µm segments (one inner and two outer segments). Magnification is × 10.5, and scale bar is 100 µm. (**B**) Inverse correlations between the myoblast:fibroblast ratio and the migration dynamics of 12 different primary muscle stem cells 24 h (P = 0.011) and a trend 48 h (P = 0.064) after the artificial wound healing assay. This figure (**B**) was produced in Microsoft Excel 2016.

However, there was an inverse correlation between myoblast:fibroblast ratio and migration dynamics for the 12 participants (Fig. 4). Muscle stem cells with a low myoblast:fibroblast ratio demonstrated more cells in the inner segment than to the outer segment compared to muscle stem cells with high myoblast:fibroblast ratio at 24 h (R^2^ = 0.49, F_1,10_ = 9.53, P = 0.011) and with a non-significant trend at 48 h (R^2^ = 0.30, F_1,10_ = 4.33, P = 0.064) after the artificial wound healing assay reinforcing the preliminary in vitro results from our laboratory.

### Comparison of the muscle response between the repeated maximal sprint protocol and the muscle stem cell study

**Figure 5:**
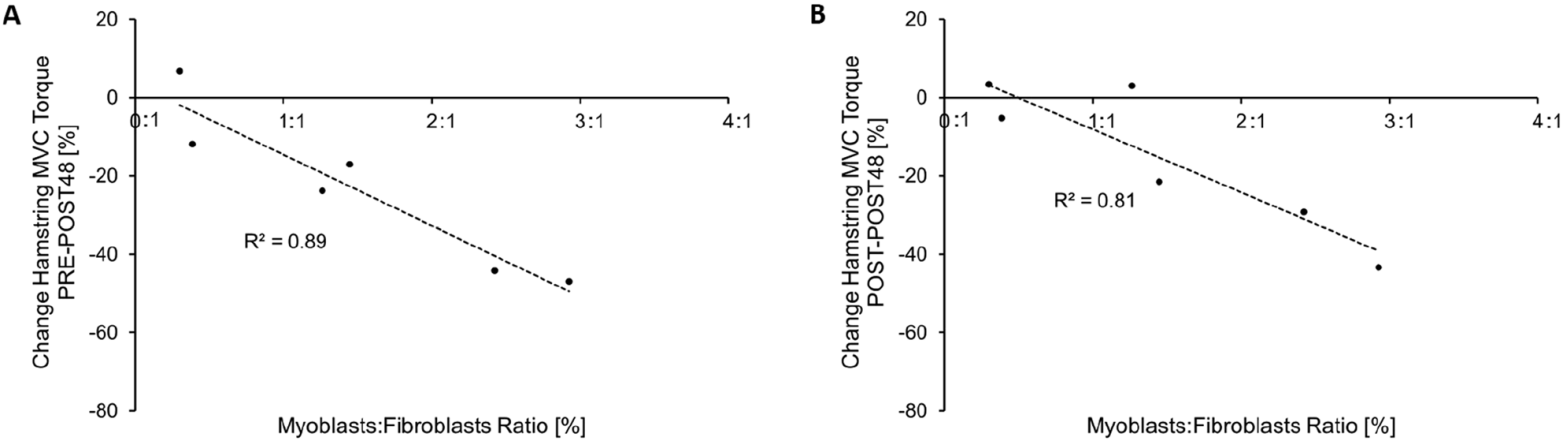
Inverse correlation between the myoblast:fibroblast ratio, assessed in the current in vitro study and the change of hamstring MVC torque measured (**A**) before and 48 h after (P = 0.004), and (**B**) measured immediately after and 48 h after (P = 0.014) (**B**) the repeated maximal sprint intervention. This figure was produced in Microsoft Excel 2016.

In order to determine whether skeletal muscle stem cell ratio (i.e. myoblast:fibroblast ratio) played a role in muscle strength recovery in vivo, further studies were performed. Previous investigations have shown that skeletal muscles of different origin, but with similar physiological functions and fibre type composition demonstrate similar transcriptome expression patterns of up to 99%^32,33^. Further, all limb muscles arise developmentally from the ventrolateral dermomyotome of the segmented paraxial mesoderm^34^ and these muscles with a similar fibre type composition show low intra-subject^35,36^ but high inter-subject variability^29,36^ regarding the total amount of stem cells within these muscle fibres. Thus, muscle biopsies were obtained from the vastus lateralis of six participants, who also performed the repeated maximal sprint intervention (they volunteered to provide a muscle biopsy at least three weeks before completing the repeated maximal sprint intervention). As muscle fibre-type composition is similar between the quadriceps and hamstrings^32^, the stem cell composition of the vastus lateralis was considered representative of both the quadriceps and hamstring muscle groups. The mean ± SD myoblast:fibroblast ratio of the six participants was 1.46 ± 1.06 (range 0.299–2.93). There was an inverse correlation between myoblast:fibroblast ratio and the percentage change in relative hamstring MVC torque measured PRE-to-POST48 in vivo (R^2^ = -0.89, F_1,4_ = 33.73, P = 0.004; Fig. 5). Thus, participants with a high myoblast:fibroblast ratio showed a delayed hamstring strength recovery 48 h after repeated maximal sprints compared to those with a low myoblast:fibroblast ratio. Further, there was an inverse correlation between myoblast:fibroblast ratio and relative hamstring MVC torque measured POST-to-POST48 (R^2^ = − 0.81, F_1,4_ = 17.08, P = 0.014; Fig. 5). No correlations were found between the myoblast:fibroblast ratio and changes in quadriceps MVC torque or with any other muscle damage and fatigue biomarker following repeated maximal sprints. These inverse correlations could be confounded by the model assumptions and the relative low participant number. However, post hoc power calculations demonstrate that the bivariate correlation had adequate power (0.97).

## Discussion

**Figure 6:**
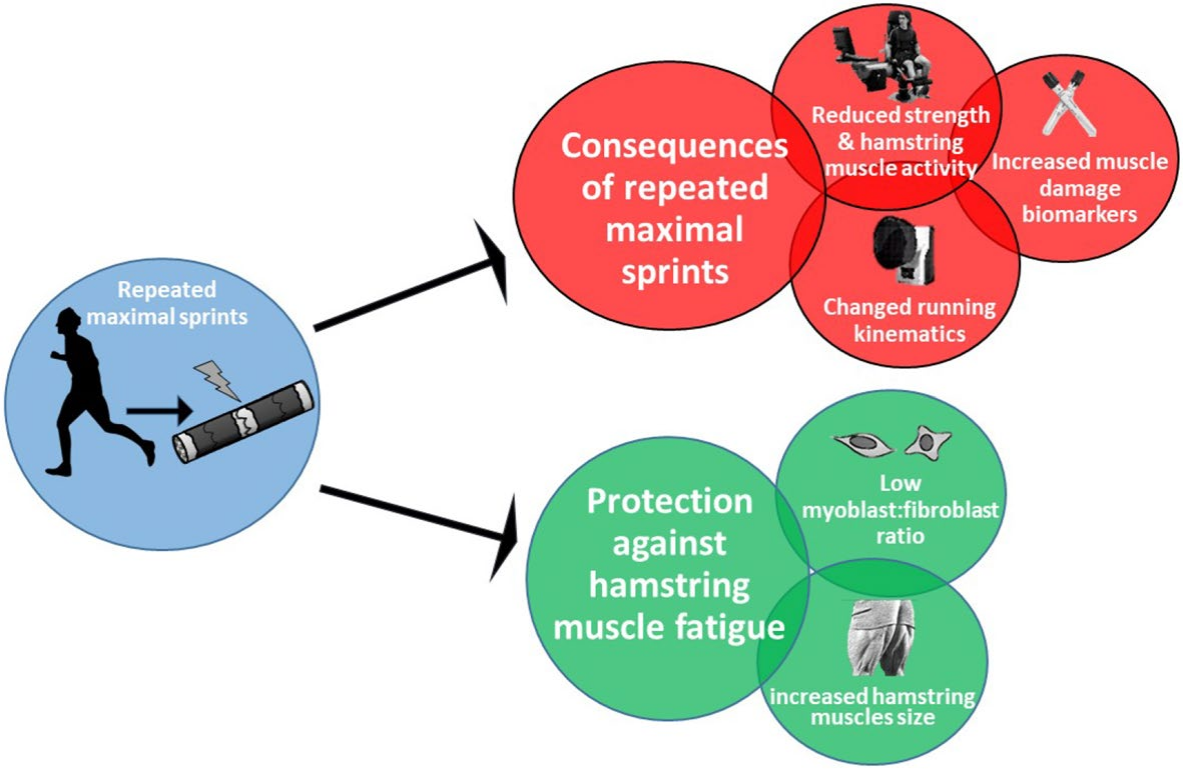
Summary figure describing the main findings of the current paper.

In this study, we have used an interdisciplinary approach to investigate the potential biomechanical, physiological and cellular factors underpinning neuromuscular fatigue following repeated maximal sprints. We have shown that immediate strength loss was associated with reduced hamstring sEMG activity (indicating impaired hamstring motor unit recruitment) and markers of peripheral fatigue, but the magnitude and sustained changes in MVC torque over time (especially in the hamstrings) was largely associated with indicators of peripheral fatigue. Muscle damage biomarkers indicated that the hamstring peripheral fatigue might have been caused predominantly by ultrastructural damage within the muscle tissue. Further, both central and peripheral fatigue caused by repeated maximal sprints appeared to affect the neuromuscular control of running patterns, while a larger BF_LH_ PCSA was related to attenuated hamstring strength loss immediately after the repeated maximal sprint intervention. Finally, our results suggest that a high myoblast:fibroblast ratio leads to a delayed wound closure in vitro and to a delayed MVC torque recovery following repeated maximal sprints in vivo within the first 48 h, indicating that stem cells of the non-contractile muscle tissue might positively affect the response to muscle damaging exercise. A visual summary of the main findings of this manuscript are depicted in Fig. 6.

### Fatigue and muscle damage following repeated maximal sprints

We showed a decreased activity of normalised BF_LH_ sEMG activity immediately after the repeated maximal sprint intervention, but no significant changes in neuromuscular activation using the interpolated twitch technique. The discrepancy between these two methods might be explained by the fact that voluntary activation measured via the interpolated twitch technique investigates all of the hamstring muscles, whilst the normalised EMG analysis was confined solely to the BF_LH_, which is in line with a previous study^5^. The BF_LH_ might fatigue to a greater degree immediately after repeated maximal sprint related interventions compared to the other hamstring muscles. However, we also provide evidence for peripheral fatigue occurring immediately after the repeated maximal sprints and a delayed recovery at higher frequencies (30–50 Hz) after observing a right shift in the torque-frequency relationship. This may be due to ultrastructural damage predominantly in fast-twitch (which fire at rates from 30 to 50 Hz) compared to slow-twitch muscle fibres (discharge rates 10–25 Hz)^37,38^, leading to impaired force generation rather than simply fatiguing the muscle fibres.

Both the quadriceps and hamstring muscle groups showed similar strength loss immediately after the repeated maximal sprints, but the hamstring muscle group showed further strength loss 48 h later compared to the quadriceps. Other studies did not show this additional strength loss for the hamstring muscle group POST48. Differences in the training status of the participants^7,25^ and in the methodological approaches^39^ might partly explain the different outcomes. The peak BF_LH_ EMG activity occurs at a more extended knee angle during hamstring isokinetic muscle contraction compared to the peak EMG occurring at a more flexed knee angle for the other hamstring muscles, such as the semitendinosus^40^. Therefore, we suggest that the BF_LH_ is the key hamstring muscle responsible for decelerating the shaft at the end of the late swing phase. After repeated bouts of high-speed running, the semitendinosus might fatigue prematurely^41^ and the BF_LH_ would need to substitute the impaired function of the preceding semitendinosus to decelerate the shaft.

During high-speed running, eccentric contractions occur in the hamstrings during the late swing phase (i.e. during deceleration of the knee extension)^42^ and in the quadriceps during the early/mid swing phase (= deceleration of hip extension)^43^. In general, the likelihood of sustaining a quadriceps strain injury is significantly lower compared to a hamstring strain injury^2^. If repeated eccentric contractions are one of the main causes for strain injuries, as indicated by several investigations^44,45^, then the difference in strength loss between the quadriceps and hamstrings observed in our study might be explained by the different levels of eccentric force generated by the muscles. Muscle damage in the quadriceps muscle presumably occurs during the deceleration phase of sprinting and during the backswing phase, when the quadriceps muscle works eccentrically to decelerate the leg with a flexed knee during the early swing phase of high-speed running^26,43^. Hamstring strain injury, however, occurs with an almost extended leg during the late swing phase^42^. This extended lever arm might cause higher eccentric force in the hamstring muscles compared to the shorter lever arm with a flexed knee on the quadriceps muscle. Continuously repeated eccentric contractions with the longer lever arm will potentially induce more muscle damage within the hamstrings in total during e.g. a soccer match, compared to the quadriceps muscle group, which might explain the significant different strength loss POST48 repeated sprints in the current study, and potentially the different injury rates between these two muscle groups.

### Kinematic analysis

Our in vivo intervention caused changes in the running kinematics with reduced knee extension in the late swing phase immediately after the repeated maximal sprints. Reduced hamstring muscle strength due to neuromuscular fatigue might trigger a protective mechanism directly after repeated maximal sprints. Afferent signals from the fatigued and damaged hamstrings might activate the Golgi tendon organ^4^, thus limiting hamstring muscle fibre strain in an attempt to minimise further muscle damage. These kinematic changes were not evident 48 h after the repeated maximal sprints. However, there was a non-significant tendency for prolonged stride duration during running (P = 0.08, data not shown) 48 h later and the percentage change of knee extension in the late swing phase of running correlated with changes in hamstring strength both measured from POST to POST48. This indicates that participants with delayed hamstring strength recovery were still not able to fully control running. As hamstring MVC continued to deteriorate 48 h after repeated maximal sprints but quadriceps MVC started to improve, it could be that lower-limb kinematics in the sagittal plane are controlled by the hamstrings more than the quadriceps. That outcome could also have implications for the underlying mechanisms of knee injuries, such as an anterior cruciate ligament injury^46^, which would need further investigation. Summarised, ultrastructural damage in the hamstring muscles might lead to decelerated movement patterns over time, which could increase the risk for hamstring strain injury during sprinting^3^.

### The role of the extracellular matrix on the muscle response following repeated maximal sprinting

Recent investigations have suggested that hamstring maximum eccentric strength and BF_LH_ fascicle length are predictors of hamstring strain injury^22^. Further, a fatigued muscle is likely to accentuate the risk of muscle strain^2^. However, we could not find any correlation between BF_LH_ fascicle length and any biomarker of fatigue but BF_LH_ PCSA correlated inversely with hamstring strength loss from PRE to POST. During the late swing phase of sprinting, the hamstring muscles contract eccentrically to decelerate the shaft and to enhance the subsequent concentring shortening contraction for maximal sprinting by using stored elastic energy from the muscle–tendon unit. In comparison to other conventional muscle-damaging interventions^47^, this dynamic (stretch–shortening) movement might lead to an additional damage of the hamstring muscle connective tissue structure. Therefore, a larger BF_LH_ PCSA might protect against immediate hamstring MVC loss due to a greater ability to transmit the ground reaction forces laterally (from fibre to fibre)^48^, which might disperse the force more efficiently to the tendon, while the muscle fibres themselves undergo less strain. Further, a greater BF_LH_ PCSA reflects more fibres aligned in parallel, which would be accompanied by more muscle connective tissue of the extracellular matrix, thus potentially protecting the muscle fibres from excessive damage during eccentric contractions.

The stem cells of the extracellular matrix also demonstrated an important role for muscle strength recovery in the subgroup of participants, as there was a strong inverse correlation between myoblast:fibroblast ratio and hamstring MVC torque recovery POST48. Skeletal muscles with a higher availability of fibroblasts around the area of myotrauma might have a better capacity to reorganise the complex extracellular matrix, thus restoring (lateral) force transmission, which results in a faster recovery of muscle strength after muscle damage. This was in line with the myoblast:fibroblast ratio effect on cellular aspects of muscle regeneration and remodelling assessed in primary muscle stem cells in vitro. Muscle stem cells with a low myoblast:fibroblast ratio revealed a faster wound closure (i.e. more cells migrated to the inner part of the artificial injury compared to the outer part), in particular 24 h after performing the scratch assay. Other research with a co-culture assay (myoblasts and fibroblasts were physically separated but shared the same supernatant/media to investigate fibroblast-secreted factors) also observed that a higher number of fibroblasts increase myoblast migration into an artificial wound^49^. Thus, the interaction between myoblasts and a larger abundance of fibroblasts near the micro trauma of the muscle seems to have a positive effect on healthy muscle regeneration at the beginning of muscle repair.

The contribution of fibroblasts to the early phase of muscle repair has previously been investigated in animal studies in vivo^15^. The amount of muscle ECM increased to peak levels three days after a barium chloride-induced injury in mice. This was accompanied with rapid proliferation of muscle fibroblasts in close proximity to satellite cells and both the amount of ECM and of muscle fibroblasts returned to baseline levels 21 days after the injury. Transgenic mice with Pax7^+^ satellite cells deficiency showed dramatically impaired muscle regeneration from day five onwards, and generated fibrosis after the chemically mediated injury. Further, genetically engineered mice with Tcf4^+^ fibroblast deficiency demonstrated a premature activation and differentiation of Pax7^+^ satellite cells three days after the barium chloride-induced injury, which led to a decreased number of satellite cells over the following days.

Recent human in vivo investigations confirmed the interdependence of muscle fibroblasts and satellite cells for a healthy muscle regeneration^14^. However, the time frame of muscle fibroblast accumulation around the regenerating muscle fibres happened at a later time point (30 days) following electrical stimulations compared to Murphy, et al.^15^. Presumably, the different results are based on the different organisms investigated, varying (parts of) muscles studied and different injury protocols used. It can be assumed that involuntary isometric contractions induced by artificial electrical stimulation potentially damage more proteins, which anchor the actin filaments at the Z-line^50^. However, these involuntary isometric contractions might damage the muscle ECM (particularly the perimysium) to a lesser extent compared to physiological eccentric MVCs^50^, dynamic (stretch–shortening) movements (such as in the current study) or barium chloride-induced injury^51^. That might explain the delayed fibroblast proliferation^14^ at the early stage of muscle repair in involuntary isometric contractions compared to barium chloride-induced injury in mice^15^.

However, our study showed that the effect of the myoblast:fibroblast ratio was less significant 48 h after the scratch protocol. As the master regulator of collagen biosynthesis Rrbp1 is suppressed in the days after muscle damage to avoid long lasting unfavourable fibrosis^19,20^, we suggest that the abundance and activity of fibroblasts and myoblasts play different roles, depending on the time points during muscle repair, and that a larger abundance of fibroblasts has a positive effect at the beginning of muscle repair. Future in vitro studies need to investigate whether muscle-derived primary cells with a higher myoblast:fibroblast ratio would reveal improved muscle regeneration beyond 48 h, which would be in line with the in vivo investigation in mice of Fry et al.^52^. We, therefore, assume that repeated maximal sprints with insufficient recovery of previously fatigued and damaged muscles (where the fatigue and damage response is modulated by the muscle size and stem cell composition, respectively) might augment the risk of muscle strains, as appropriate damage to the muscle connective tissue is thought to differentiate between exercise-induced muscle damage and muscle strains^53,54^.

The practical implications of our study are that a 48 h recovery period following repeated maximal sprinting is insufficient, and might increase hamstring strain injury risk. Furthermore, increasing hamstring PCSA via resistance training is likely to reduce peripheral fatigue following repeated maximal sprinting, thereby reducing hamstring strain injury risk.

## Limitations

The current study observed a relationship between human primary muscle cell type (in vitro) and physiological biomarkers of skeletal muscle damage/recovery following strenuous exercise. Further research is necessary to confirm these results with a larger sample size regarding the in vitro study. However, given the scarcity of data that have examined human muscle stem cell characteristics in association with muscle damage/recovery in vivo, and that most of our results were sufficiently powered, we believe that our study represents an important advancement in our understanding of how skeletal muscle recovers following strenuous exercise. There was no relationship between the myoblast:fibroblast ratio and any physiological variables regarding the quadriceps femoris, from which the muscle biopsies were obtained. It has previously been shown that skeletal muscles of different origin, but with similar physiological functions and fibre type composition, demonstrate similar transcriptome expression patterns of up to 99%^32,33^. For immnunohistochemistry analysis, the ICC (3, k) of 0.83 (95% Cis 0.59–0.95) indicates a good reliability for the characterisation and the quantification of myoblasts and fibroblasts. Therefore, it is likely, that the correlation between myoblast:fibroblast ratio and the muscle damage-response of the hamstrings but not the quadriceps muscles is explained by more severe ultrastructural damage in the hamstrings than quadriceps. With our in vitro study design, we reported an inverse correlation between the myoblast:fibroblast ratio and migration dynamics. However, we cannot assume that this inverse correlation implies causation, i.e. we cannot confirm whether the myoblast:fibroblast ratio was the factor that led to lower numbers of migrating cells in high versus low myoblast:fibroblast ratio, or whether the observed difference was due to individual differences in the intrinsic capacity for migration of these skeletal muscle stem cells obtained from these different participants. In addition, we cannot completely exclude proliferation during the in vitro wound healing assay (particularly in the outer wound edge), which might impact the data (resulting in an under-estimate of the percentage of cells migrating to the centre of the wound). However, previous data from our laboratory^29,55^ with mitomycin-c, real time movies of migration, and the fact that cell proliferation in low serum medium is arrested to facilitate fusion, suggest that this is unlikely. Furthermore, peripheral fatigue can be caused by metabolic perturbations, such as the depletion of intramuscular glycogen^12^. Therefore, because we did not control diet throughout the study, it is possible that inter-individual differences in baseline muscle glycogen may have influenced the ability to maintain maximal intensity throughout the sprints. However, participants were instructed to eat and drink similar foods two hours before each laboratory visit, and to avoid strenuous exercise for at least 48 h prior to the testing. Further, participants were given sufficient recovery between sprint repetitions and there was a low decrement in sprint performance, indicating that glycogen depletion was probably only a minor factor.

## Conclusion

Repeated maximal sprints induce a greater and more prolonged strength loss in the hamstrings compared to the quadriceps muscles. The immediate loss of hamstring function appears to be due to both central (particularly reduced neuromuscular activation of the biceps femoris long head) and peripheral fatigue, while prolonged hamstring strength loss is predominantly linked to peripheral fatigue. Thigh neuromuscular fatigue following repeated maximal sprints alters hip and knee kinematics during running immediately after the repeated maximal sprints, which may lead to an increased hamstring muscle injury risk. Furthermore, biceps femoris long head PCSA was inversely related to hamstring strength loss immediately after repeated maximal sprinting. This suggests a greater PCSA may help transmit more ground reaction force laterally between muscle fibres, e.g. via the extracellular matrix, thus placing less stress during eccentric contractions on the individual muscle fibres and protecting against muscle damage/fatigue. Furthermore, our results suggest that skeletal muscles with an increased number of fibroblasts might have a better capacity to reorganise the complex extracellular matrix, which results in a faster wound closure after substantial muscle damage.

## Materials and methods

A full account of the Methods can be found in the “Supplementary information”, ‘Full Methods’. The following section summarises the methods used in this study.

### Participants

Twenty recreationally active and healthy young men (mean ± SD; age 20.3 ± 2.9 years; body mass 75.0 ± 7.9 kg) participated in the repeated maximal sprint intervention. Twelve healthy young male and females (age 22.8 ± 4.0 years; body mass 68.9 ± 7.1 kg) provided a biopsy of the vastus lateralis muscle for the in vitro muscle stem cell component of this study*.*

Prior to starting the study, written informed consent was obtained from each participant and pre-biopsy screening was performed by a physician for those participants who volunteered a muscle biopsy. The study was approved by the Research Ethics Committee of Liverpool John Moores University and complied with the Declaration of Helsinki.

### Experimental design of the repeated maximal sprint intervention in vivo

One week prior to the testing day, participants were familiarised with the assessments. On the test day, participants performed the repeated maximal sprint intervention of 15 × 30 m sprints with a deceleration zone of 12 m to induce neuromuscular fatigue/damage in both the quadriceps femoris and hamstring muscle groups. The recovery comprised 90 s between repetitions and after every 5th repetition, the participants were allowed to rest for 3 min. The test battery was performed PRE, POST and POST48 following the repeated maximal sprint intervention.

### Maximal voluntary contraction (MVC)

We tested *isometric* MVC quadriceps (at 80° knee flexion; 0° = full knee extension), and hamstring torque (at 30° knee flexion) with an isokinetic dynamometer (Humac Norm, CSMI Solutions, Massachusetts, USA). The participant was seated in an upright position and the hip joint angle was set to 85° (180° = supine position). Participants performed three isometric knee extension (quadriceps) and flexion (hamstring) at both joint angles (each MVC lasting 2–3 s), with 60 s rest between MVC of a given muscle group.

### Hamstring muscle voluntary activation

To measure hamstring muscle voluntary activation capacity via the interpolated twitch technique, stimulation electrodes (12.5 mm × 7.5 mm self-adhesive electrodes (DJO Global, California, USA) were used. The anode was placed proximal to the popliteal fossa, and the cathode was placed beneath the gluteal fold and slightly medial to avoid activation of the vastus lateralis. Stimulation was delivered by a high-voltage stimulator (DS7AH; Digitimer Ltd., Welwyn Garden City, United Kingdom), and consisted of a doublet using two 240-V rectangular pulses (200 µs pulse width) with an inter-pulse duration of 10 ms (100 Hz stimulation). The maximal doublet stimulation was used two minutes later to elicit resting maximal doublet torque in the resting state (control doublet), followed 2.5 s later by a second (superimposed) doublet during an isometric knee flexion MVC. Voluntary activation was calculated according to the following equation:

$$ VA\left( \% \right) = \left[ {1 - \left( {superimposed \, doublet \, torque/control \, doublet \, torque} \right)} \right] $$

### Surface electromyography and antagonist muscle co-activation

Surface electromyographic (sEMG) activity was recorded from the vastus lateralis and BF_LH_ to determine the extent of antagonist muscle co-activation during MVCs of the respective muscle group. Two bipolar Ag–AgCl surface electrodes (Noraxon duel sEMG electrode, Noraxon, Scottsdale, USA) were placed along the sagittal axis over the muscle belly at 33% of the respective muscle length from the distal end and one reference electrode was positioned over the medial tibial condyle. Surface EMG activity of both the agonist and antagonist muscles were analysed by calculating the root mean square of the sEMG signal of a 500-ms epoch around peak MVC. To compare BF_LH_ sEMG activity at all three time points, BF_LH_ sEMG was normalised to the evoked maximum compound muscle action potential (M-wave) of the BF_LH,_ and antagonist muscle co-activation was calculated with the following formula (EMG_max_ is the maximum sEMG of the antagonist muscle when acting as an agonist at the same knee joint angle):

$$ Antagonist\;muscle\;co{\text{-}}activation = {\frac{EMG_{antagonist}}{{EMG_{max}}}} \times 100 $$

### Hamstring muscle maximal compound muscle action potential

The hamstring muscle group was stimulated with single square wave twitch pulses (200 µs duration). While the participant sat resting on the isokinetic dynamometer with the knee angle set at 30° knee flexion, compound muscle action potentials (*M*-waves) were evoked with 10–20 mA incremental amplitudes until a maximal *M*-wave (*M*_max_) was achieved. The *M*_max_ was defined as the mean peak-to-peak sEMG response from the three highest observed *M*-waves. We normalised absolute BF_LH_ sEMG to the individual’s BF_LH_ *M*_max_.

### Torque-frequency relationship

The torque-frequency relationship was determined by stimulating the hamstring muscle group with single square wave twitch pulses (200 µs duration) at 1, 10, 15, 20, 30, 50 and 100 Hz for 1 s each in a random order and with 15 s rest between each stimulation. The stimulus intensity for 100-Hz stimulation was the amplitude necessary to elicit ~ 20% knee flexion MVC torque at PRE, and the same amplitude was used for the same test at POST and 48POST. The absolute peak torque at each frequency was normalised to the peak torque at 100 Hz for each time point.

### Ultrasound

Architectural parameters of the BF_LH_ were assessed using B-mode ultrasound imaging. Participants were in the prone position with the hip and knee fully extended and muscles relaxed. Longitudinal (incorporating the intra-muscular aponeurosis; Fig. 3) and cross-sectional panoramic ultrasound images at 20, 40, 60 and 80% along the total muscle length (Fig. 3) of the right BF_LH_ were obtained (Philips EPIQ 7 Ultrasound System, Bothel, USA). The volume of the muscular portion between every two consecutive scans was calculated with the following equation:

$$Volume=\frac{1 }{3 }*d*\left(a+\sqrt{\left(ab\right)+b}\right)$$

where *a* and *b* are the anatomical cross-sectional areas of the muscle of two consecutive cross-sectional scans and *d* is the interval distance between the cross-sectional area measurements. Resting BF_LH_ muscle fascicle length and pennation angle were both assessed in three fascicles at 50% of the total length of BF_LH_.

### Kinematic and kinetic data

Three-dimensional kinematic and kinetic data were synchronously collected at 500 Hz using an eight-camera motion analysis system (Qqus 300+; Qualisys, Gothenburg, Sweden). Retroreflective markers (12 mm diameter) were placed on anatomical landmarks on the right leg and pelvis. Kinematic data were tracked using Qualisys Track Manager Software (Qualisys). Data processing and analysis were undertaken in Visual3D (C-Motion, Germantown, MD). Lower extremity 3D joint angles and angular velocities were calculated using an X–Y–Z Cardan angle rotation sequence. Participants ran on a motorised treadmill (HP Cosmos Pulsar; Nussdorf, Germany) for 30 s at 4.17 m s^−1^ (0° incline), and motion analysis data were recorded for the last 10 s of the run and for at least six consecutive strides.

### Blood samples

A 10 mL blood sample was drawn from an antecubital vein in the forearm and collected into a serum vacutainer (BD Vacutainer systems, Plymouth, UK). The blood samples were obtained at each time point and left at 22–24 °C for 30 min to allow clotting, and then kept on ice when necessary. Serum samples were centrifuged at 1300*g* for 15 min at 4 °C. All samples were then aliquoted into 1.5 mL microcentrifuge tubes [Axygen (Corning), New York, USA] and stored at − 80 °C until subsequent analysis.

### Serum interleukin-6 (IL-6) concentration and creatine kinase activity

Serum samples were assayed for IL-6 concentration using commercially available human IL-6 enzyme linked immunosorbent assay kits (Quantikine, R&D systems, Minneapolis, MN, USA) according to the manufacturer's instructions. Creatine kinase (CK) activity was assayed using a commercially available CK assay (Catachem Inc., Connecticut, NE, USA). Ten μL blood serum were loaded onto a 96-well plate. The CK reaction reagent and diluent (Catachem) were prepared as per the manufacturer’s instructions and added to the samples and the change in absorbance monitored continuously over 20 min in a Thermo Multiskan Spectrum plate reader (Thermo Fisher Scientific. Waltham, MA. USA) at a wavelength of 450 nm (IL-6) and of 340 nm (CK activity).

### Capillary blood lactate concentration

Capillary blood samples were taken from the finger-tip via a Safety-Lancet Extra 18G needle (Sarstedt; Nümbrecht, Germany) at rest before and immediately after the repeated maximal sprint intervention using a portable blood lactate analyser (Arkray Lactate Pro; Kyoto, Japan).

### Muscle biopsy procedure and extraction of human muscle-derived cells

Biopsies from the vastus lateralis muscle were obtained under local anaesthesia from each participant, using the Weil-Blakesley conchotome technique. The muscle biopsies analysed in this study were isolated and cultured^29^, as reported previously. Briefly, biopsy samples were cut in small pieces (1 mm^3^) and digested in 5 mL of trypsin–EDTA for 3 × 15 min on a magnetic stirring platform at 37 °C to dissociate muscle-derived mononuclear cells. Supernatant derived following each treatment was collected and pooled with hiFBS. Cell supernatant was centrifuged at 450*g* for 5 min. Cell pellet was resuspended in growth media [Hams F-10 nutrient mix (Lonza, Basel, Switzerland) with added l-glutamine (2.5 mM), 10% heat-inactivated FBS (Gibco, Thermo Fisher Scientific, Altincham, UK), and 1% penicillin–streptomycin (Life Technologies, Warrington, UK)] and plated on a pre-coated T25 cm^2^ culture flask (Corning, Life Sciences, New York, USA) for cell population expansion. The cells were expanded until passage 3 and then frozen in GM with 10% dimethyl sulfoxide (DMSO) in liquid N_2_ as a cryopreservant.

### Characterization of human muscle-derived mononuclear cells

**Figure 7:**
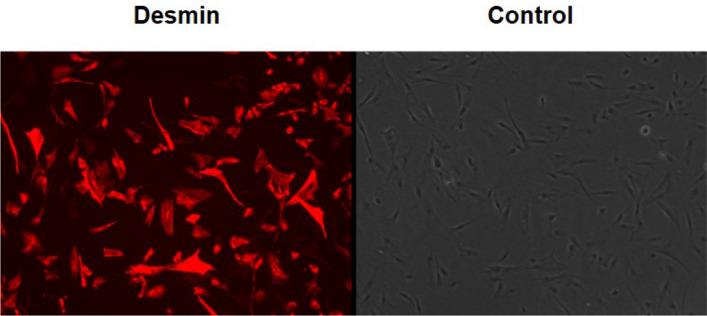
Representative images for immunofluorescent staining for desmin of human muscle cell cultures. Myoblasts are desmin positive (red) and non-myoblasts are desmin negative. Myoblast to fibroblast ratio is 2.9. Magnification is × 10.5.

Mononuclear cells, resident within the biopsies, were isolated and cultured in vitro, enabling determination of myoblast:fibroblast ratios (Fig. 7). To determine the percentage of myoblasts, the mixed population of human skeletal muscle-derived mononuclear cells, were characterised by immunofluorescent staining for the detection of desmin-positive myoblasts and desmin-negative cells, which is highly enriched (up to 99%) in fibroblasts^56^. Grohmann, et al.^30^ reported that passaging does not change the percentage of myoblast and fibroblasts and, therefore, all populations were included for analysis.

Monolayers were incubated with 25% [vol/vol methanol in TBS, 50% and 100% for 5-min to fix the cells and stored at 4 °C wet in TBS until further analysis. Fixed monolayers were permeabilised and blocked for 2 h with 5% goat serum and 0.2% Triton X-100 in TBS, prior to staining. Cells were incubated overnight at 4 °C with Desmin polyclonal rabbit anti-human antibody (Cat# ab15200, RRID: AB_301744) antibody (1:200; Abcam, Cambridge, UK). After overnight incubation, the primary antibody was removed, and the cells were washed three times with TBS. Secondary TRITC polyclonal goat anti-rabbit (Cat# A16101, RRID: AB_2534775) ] antibody (1:200; Fisher Scientific) was then applied and incubated for 2 h at 4 °C. Fluorescent images were captured using live imaging microscopy (Leica DMB 6000; Magnification 10.5×) and analysed via ImageJ cell counter plug-in.

### Wound-healing assay, migration and differentiation analysis

One hundred thousand cells/mL were seeded in gelatinised six-well plates (Nunc, Roskilde, Denmark). Cells were expanded as described above until cell monolayers reached a confluent state, growth media was removed, monolayers were washed with PBS and cells were damaged by a vertical scrape with a 1-mL pipette tip, as previously reported by our group^29^. PBS was aspirated, damaged cell monolayers were washed twice with PBS and 2 mL media with reduced FBS (2%) was added. Monolayers were imaged with a live imaging microscopy (Leica) for the analysis of cell migration into the wound site, immediately, 24 h, and 48 h. TIF images were exported from Leica Application Suite and loaded as TIF image stacks in ImageJ with a cell counter plug-in. Cells in the outer and inner segments were then counted (Fig. 4), and analysed in ImageJ.

### Statistical analysis

One-way repeated-measures analysis of variances (ANOVAs) were performed to determine whether there was a significant main effect for time (within subject factor). MVC torque data were analysed for interactions and main effects for muscle group and time using two-way mixed design ANOVAs. For within test comparisons, either independent t-tests, or one-way ANOVAs were used where appropriate. For the torque-frequency relationship, normalised torque at each frequency was analysed using a two-way repeated measures ANOVA. Post-hoc one-way repeated measures ANOVAs were used to determine if the normalised torque at each frequency differed between time points. Bivariate correlations were used to analyse the relation between architectural parameters of the BF_LH_ and fatigue biomarkers as well as between myoblast:fibroblast ratio and quadriceps and hamstring MVC, and migration dynamics of the muscle stem cells. Results were expressed as mean ± SD, unless otherwise stated, with statistical significance set at P < 0.05. All MVC, sEMG and ITT data were analysed with AcqKnowledge software 4.4 (Biopac-Systems Inc., Goleta, USA) and SPSS 23 Software (IBM Inc., Armonk, NY: IBM Corp).

## Supplementary Information

Supplementary Information 1.

Supplementary Information 2.

## Data Availability

The datasets that support the findings of this study are available as “Supplementary Information” files.
