# Supplementary material for: Neuromuscular fatigue and recovery after strenuous exercise depends on skeletal muscle size and stem cell characteristics
Source: Sci Rep. 2021 Apr 8;11:7733. doi: 10.1038/s41598-021-87195-x (PMC8032692; doi:10.1038/s41598-021-87195-x)
Supplement: Supplementary file 2 — Supplementary Information 2. [file 41598_2021_87195_MOESM2_ESM.pdf]

Supplementary Information (SI) for: Neuromuscular fatigue and recovery after strenuous exercise depends on skeletal muscle size and stem cell characteristics

Baumert, P.[1,2]*, Temple, S.[2], Stanley, J.M.[2], Cocks M. [2], Strauss, J.A. [2], Shepherd S.O.[2], Drust, B.[3], Lake, M.J.[2], Stewart, C.E.[2], Erskine, R.M.[2,4]

[1] *Exercise Biology Group, Faculty of Sport and Health Sciences, Technical University of Munich, Munich, Germany*

[2]*Research Institute for Sport & Exercise Sciences, Liverpool John Moores University, Liverpool, United Kingdom.*

[3]*School of Sport, Exercise and Rehabilitation Sciences, College of Life and Environmental Sciences, University of Birmingham, Birmingham, UK.*

[4] *Institute of Sport, Exercise & Health, University College London, London, UK*

Running title: Neuromuscular Response following muscle damage *in vivo* and *in vitro*

Address for reprint requests and all other correspondence:

Philipp Baumert, Exercise Biology Group, Faculty of Sport and Health Sciences, Technical University of Munich, Munich, Germany; Email: philipp.baumert@tum.de

SUPPLEMENTARY FULL METHODS

S1 Participants

In vivo repeated maximal sprint intervention: Twenty recreationally active and healthy young men (*mean* ± *SD*; age 20.3 ± 2.9 years; height 1.79 ± 0.05 m; body mass 75.0 ± 7.9 kg) participated in the repeated maximal sprint intervention.

In vitro muscle stem cell component: Eight healthy young male (age 21.25 ± 4.27 years; height 1.77 ± 0.05 m; body mass 73.78 ± 5.68 kg) and four healthy young female (age 25.5 ± 1.29 years; height 1.67 ± 0.08 m; body mass 61.40 ± 2.57 kg) participants provided a biopsy of the vastus lateralis muscle for the *in vitro* muscle stem cell component of this study. Six of the eight males also participated in the repeated maximal sprint intervention at least three weeks after providing a muscle biopsy. Only males were recruited for the repeated maximal sprint intervention, as there is some evidence of sex differences in neuromuscular fatigue [1]. However, both men and women were recruited for the muscle stem cell component due to there being no reported sex differences in stem cell properties and none within our own pilot studies (data not shown). Prior to starting the study, written informed consent was obtained from each participant and pre-biopsy screening was performed by a physician for those participants who volunteered a muscle biopsy. The study was approved by the Research Ethics Committee of Liverpool John Moores University and complied with the Declaration of Helsinki. Volunteers were physically active but were ineligible to participate if they had performed strength training of the lower limbs within 6 months prior to participation in the study, which was determined during pre-participation screening. Further exclusion criteria were: (i) any lower limb injury in the past 12 months; (ii) age under 18 or above 35 years; and (iii) more than three structured exercise sessions per week.

S2 Experimental Design of the Repeated Maximal Sprint Intervention *in vivo*

Participants were required to visit the temperature-controlled (22-24°C) laboratory on three occasions: (i) familiarisation, (ii) testing day including the PRE and POST assessments; and (iii) POST48 assessments after the repeated maximal sprint intervention. One week prior to the testing day, participants were familiarised with the assessments as well as with repeated maximal sprints (by performing 2-3 submaximal sprints) and BF_{LH} architecture of the hamstring muscle group was assessed via ultrasound. On the test day, participants performed the repeated maximal sprint intervention of 15 x 30 m sprints to induce neuromuscular fatigue/damage in both the quadriceps femoris and hamstring muscle groups. All tests were performed at the same time of the day for each participant. Further, participants were instructed to maintain their normal routine (including eating habits), to refrain from drinking alcohol and to avoid any strenuous exercise 48 h prior to testing and throughout the study, and to refrain from consuming caffeine on testing days. Nothing was consumed throughout the testing sessions except water, which was available ad libitum.

The test battery was always performed in the same order with the right leg of each participant and comprised (i) venous blood sampling [for analysing serum interleukin-6 (IL-6) concentration and creatine kinase (CK) activity]; (ii) hamstrings and quadriceps muscle soreness via visual analogue scale; (iii) isometric maximum voluntary contraction (MVC) torque of the quadriceps, as well as both voluntary and involuntary muscle activation and torque-frequency relationship via electrical stimulation] MVC torque of the hamstring together with normalised BF_{LH} sEMG (see below); and (iv) treadmill running (4.17 m s^{-1}) kinematics of the right leg (via an eight-camera motion capture system) PRE and POST48 following the repeated maximal sprint intervention. At POST, kinematic assessments were performed first followed by the aforementioned order of the assessment for practical reasons.

S3 Maximal Repeated Sprint Protocol

Many athletes in team sports are required to perform repeated maximal sprints, which are characterised by short-duration (<10 s) and relatively longer recovery times (>60 s) between maximal sprint bouts, and have a different physiological demand compared to repeated-sprint exercises with shorter recovery times (<60 s) [2,3]. Therefore, the repeated maximal sprint intervention consisted of 15 repetitions of 30 m maximal sprints with a deceleration zone of 12 m. The 30 m distance was chosen as the upper average of both the total sprinting distance (346 ± 115 m) of wide-midfielders and the mean recovery time (70.2 ± 25.1 s) between sprint bouts in soccer [2,4], which allows the athlete to maintain the performance of the sprint bouts. Similar protocols have been used elsewhere [5-7]. Prior to the repeated maximal sprint intervention, a five-minute warm-up was performed, comprising jogging, dynamic stretching and three self-paced 20 m runs at 60%, 80%, 100% of perceived top speed. During the repeated maximal sprint intervention, the participants were instructed to sprint maximally (verbal encouragement) and to stop within the deceleration zone. Further, they were instructed to move slowly back to the start line and to sit on a chair for the remaining time until the next sprint. The recovery comprised 90 s between repetitions and after every 5th repetition, the participants were allowed to rest for 3 min. Sprinting time during trials was measured and controlled with timing gates (Brower Timing Systems, Draper, UT, USA), which were placed on the start and finish line. Participants started 30 cm before the start line to avoid interfering with timing gates with the arms upon initial acceleration [8]. Further, heart rate (Polar Oy, Kempele, Finland) and rating of perceived exertion were recorded before and after each repetition. Participants were instructed to wear the same footwear for each testing day. As the fastest sprint time does not necessarily need to occur at the first of the 15 sprint bouts and there was an upsurge in speed of the final sprint, fatigue was assessed with the performance decrement score using the following formula [9]:

$$Fatigue = \left(100\,X\,(total\ sprint\ time\ \div ideal\ sprint\ time)\right) - 100$$

Where total sprint time = sum of time from all 15 sprints; and ideal sprint time = total number of sprints (15) x fastest repetition sprint time. The calculation of this decrement score was also used to quantify changes in heart rate and rating of perceived exertion during the repeated maximal sprint intervention.

S4 Maximal Voluntary Contraction (MVC)

Three test sessions were conducted with an isokinetic dynamometer (Humac Norm, CSMI Solutions, Massachusetts, USA). As *isokinetic* maximum voluntary contractions (MVC) torque tests are only weak predictors of hamstring strain injury [10], we decided to focus on *isometric* MVC quadriceps and hamstring torque at optimal knee strength angles (optimal torque-joint angle relationship, see below) to avoid further fatiguing the participants. The torque signal was interfaced with an acquisition system (AcqKnowledge, Biopac Systems, Santa Barbara, USA) for analogue-to-digital conversion and sampled at a frequency of 2 kHz. The participant was seated in an upright position and securely fastened with inextensible straps at the chest and waist while the arms were held crossed above the chest. The tibiofemoral epicondyle was aligned with the lever arm rotation axis, and the lever arm shin pad was strapped to the leg, 2 cm above the centre of the lateral malleolus. A Velcro strap secured the distal thigh just above the knee. The hip joint angle was set to 85° (180° = supine position) in order to analyse the knee flexor muscle group at a sprint specific angle associated with the late swing phase of sprinting [11]. Participants were instructed to maximally extend and flex their leg to measure knee range of motion. Quadriceps MVC was measured at 80º knee flexion (0º = full knee extension), as this is the optimal joint angle for peak quadriceps MVC in healthy young men [12]. Hamstring MVC was measured at 30º knee flexion based on this being the optimal joint angle for peak hamstring MVC during our pilot work. Published studies during the time of data collection used a similar angle of hamstring MVC torque [13,14]. This was also in line with sprinting kinematics, demonstrating that maximal hamstring muscle lengths during sprinting occur during the late swing phase when the knee is flexed between 30° and 45° [15]. Prior to isometric MVC

assessments, participants underwent a standardised warm up consisting of 10 submaximal isokinetic leg extensions ($60^{o}\cdot s^{-1}$). Participants then performed three isometric knee extension (quadriceps) and flexion (hamstring) at both joint angles (each MVC lasting 2-3 s), with 60 s rest between MVC of a given muscle group. The highest MVC of the three attempts for each muscle group at each angle was used for subsequent analyses. Throughout the tests, participants received verbal encouragement and biofeedback (MVC outputs) were projected onto a screen in front of the participant.

S5 Hamstring Muscle Voluntary Activation

To measure hamstring muscle voluntary activation capacity via the interpolated twitch technique, stimulation electrodes (12.5 mm x 7.5 mm self-adhesive electrodes (DJO Global, California, USA) were used. The general procedure has been described elsewhere [12,16,17]. Briefly, the anode was placed proximal to the popliteal fossa, and the cathode was placed beneath the gluteal fold and slightly medial to avoid activation of the vastus lateralis. Protocols were completed with electrical stimulation pads carefully taped down during the sprinting protocol and were additionally marked on the skin with a permanent marker, to ensure a precise relocation for the POST and POST48 tests. Stimulation was delivered by a high-voltage stimulator (DS7AH; Digitimer Ltd., Welwyn Garden City, United Kingdom), and consisted of a doublet using two 240-V rectangular pulses (200 μs pulse width) with an inter-pulse duration of 10 ms (100 Hz stimulation). During each experimental session, relaxed hamstring muscles were stimulated while participants were fixed in the isokinetic dynamometer with the same setting for knee flexion MVC (85º hip angle, 30 º knee flexion). The amplitude started with 50 mA to familiarise the participants to the stimulation and was gradually increased in 20 mA increments until a plateau in doublet torque was achieved. We decided to use the individual maximal stimulation (100%) intensity despite the fact that other publications used supramaximal stimulation (110-130%) [17] as we experienced lower MVC knee flexion torque output beyond 100%. That individual amplitude (162.0 ± 17.4 mA; range: 130–200 mA) was applied during all maximal contractions in the experimental session.

The maximal doublet stimulation was used two minutes later to elicit resting maximal doublet torque in the resting state (control doublet), followed 2.5 s later by a second (superimposed) doublet during an isometric knee flexion MVC. The superimposed doublet torque was always calculated manually from careful selection and inspection of the respective time periods compared to a normal increase in voluntary torque. Voluntary activation was calculated according to the following equation:

$$VA\ (\%) = [1 - (superimposed\ doublet\ torque / control\ doublet\ torque)]$$

S6 Surface Electromyography and Antagonist Muscle Co-activation

Surface electromyographic (sEMG) activity was recorded from the vastus lateralis and BF_{LH} to determine the extent of antagonist muscle co-activation during MVCs of the respective muscle group. Previous reports have shown that the vastus lateralis [18] and BF_{LH} [19] are representative muscles for the quadriceps femoris and hamstring muscle group, respectively. This procedure has been reported in detail elsewhere [18]. Briefly, once the muscles were identified via palpation, and the skin surface was shaved and cleaned with 70% ethanol, two bipolar Ag-AgCl surface electrodes with an inter-electrode distance of 2 cm (Noraxon duel sEMG electrode, Noraxon, Scottsdale, USA) were placed along the sagittal axis over the muscle belly at 33% of the respective muscle length from the distal end [according to SENIAM guidelines [20]] and one reference electrode (Ambu Blue, Ambu, Copenhagen, Denmark) was positioned over the medial tibial condyle. The exact location of the electrodes were marked on the participant's skin with a permanent marker to ensure precise electrode repositioning for the following assessments.

Surface EMG signals were sampled at 2000 Hz (Biopac Systems, Santa Barbara, USA) and then band-pass filtered between 10–500 Hz (AcqKnowledge, Biopac Systems, Santa Barbara, USA). Surface EMG activity of both the agonist and antagonist muscles were analysed by

calculating the root mean square of the sEMG signal of a 500-ms epoch around peak MVC. To compare BF_{LH} sEMG activity at all three time points, BF_{LH} sEMG of the hamstring MVC at 30° was normalised to the evoked maximum compound muscle action potential (M-wave) of the BF_{LH} (see below). Antagonist muscle co-activation (i.e. quadriceps activation during hamstring MVC at 30° knee flexion, or hamstring activation during quadriceps MVC at 80° knee flexion) was calculated with the following formula (where EMG_{max} is the maximum sEMG of the antagonist muscle when acting as an agonist at the same knee joint angle):

$$Antagonist\ muscle\ co-activation = \frac{EMGantagonist}{EMGmax} \times 100$$

Torque signals, electrical stimuli, and sEMG activity were displayed on a computer screen, interfaced with an acquisition system (AcqKnowledge, Biopac Systems, Santa Barbara, USA) used for analogue-to-digital conversion. Due to technical issues, co-activation data were available for hamstring n = 12; and quadriceps n=10.

S7 Hamstring Muscle Maximal Compound Muscle Action Potential

The hamstring muscle group was stimulated with single square wave twitch pulses (200 µs duration) using the same electrical stimulator and stimulating electrodes, as described above. While the participant sat resting on the isokinetic dynamometer with the knee angle set at 30° knee flexion, compound muscle action potentials (*M*-waves) were evoked with 10 to 20 mA incremental amplitudes until a maximal *M*-wave (M_{max}) was achieved. The average amplitude necessary to evoke a maximal *M*-wave was 166.8 ± 19.8 mA; range: 130–210 mA). The maximal *M*-wave was defined as the mean peak-to-peak sEMG response from the three highest observed *M*-waves. Due to inter-individual differences in subcutaneous fat and the (re)location of small sEMG electrodes over a relatively large muscle belly, sEMG amplitude is notoriously variable [21]. To reduce this inter- and intra-individual variability, we normalised absolute BF_{LH} sEMG to the individual's BF_{LH} maximal *M*-wave, determined at each testing session [22]. Due to technical issues, data of BF_{LH} sEMG normalised to maximal *M*-wave was only available for n = 13.

S8 Torque-frequency Relationship

The torque-frequency relationship was determined by stimulating the hamstring muscle group with single square wave twitch pulses (200 µs duration) at 1, 10, 15, 20, 30, 50 and 100 Hz for 1 s each in a random order and with 15 s rest between each stimulation, using the same electrical stimulator and stimulating electrodes (and location), as described above. The stimulus intensity for 100-Hz stimulation was the amplitude necessary to elicit ~20% knee flexion MVC torque at PRE, and the same amplitude was used for the same test at POST and 48POST. The absolute peak torque at each frequency was normalised to the peak torque at 100 Hz for each time point (PRE, POST and POST48).

S9 Delayed Onset Muscle Soreness

Using a visual analogue scale that consisted of a 100 mm line (scale 0-10 cm; 0 cm=no soreness; 10 cm= unbearably painful), in conjunction with both a three-repetition bilateral squat (predominantly to determine quadriceps femoris muscle soreness) [23] and lunges (predominantly to determine hamstring muscle soreness), participants rated their perceived lower limb muscle soreness along the muscle length immediately after each movement. Muscle soreness was also measured by recording the force required to elicit tenderness at nine fixed sites on the skin over the quadriceps (distal, central and proximal locations of the three superficial quadriceps heads, vastus lateralis, vastus medialis and rectus femoris) and six sites on the hamstrings (distal, central and proximal locations of both BF_{LH} and the medial hamstrings), which were previously marked with a permanent marker to ensure the same measuring position PRE, POST and POST48. At each site, a gradually increasing force was applied by the investigator with an algometer (FPK/FPN Mechanical Algometer, Wagner Instruments, Greenwich, USA) with a maximum of 10 kg/cm^2. Lying in the prone position with

the hip and knee fully extended and muscles relaxed, the participant was asked to indicate when the sensation of pressure changed to discomfort, and the force at that point was recorded.

S10 Ultrasound

Architectural parameters of the BF_{LH} were assessed using B-mode ultrasound imaging. Participants were in the prone position with the hip and knee fully extended and muscles relaxed. The BF_{LH} was investigated, as this muscle is the most commonly injured hamstring muscle in team sports, particularly during sprinting [24]. Longitudinal and cross-sectional panoramic ultrasound images of the right BF_{LH} were obtained (Philips EPIQ 7 Ultrasound System, Bothel, USA). The linear transducer (5-18 MHz; aperture 38.9 mm) was carefully placed on the skin with transmission gel and BF_{LH} was scanned (i) longitudinally from its distal (=0% muscle length) to proximal (=100% muscle length) myotendinous junction along a line drawn with a permanent marker to mark the central pathway between the medial and lateral aspects of the muscle (incorporating the intra-muscular aponeurosis; and (ii) cross-sectionally at 20, 40, 60 and 80% along the total muscle length, measured on the skin using a tape measure (Seca, Hamburg, Germany) (Figure 2).

All images were analysed offline (ImageJ, version 1.51s, National Institutes of Health, Bethesda, USA). Two images for each of the four cross-sectional points were recorded and the image of best quality was used to calculate BF_{LH} muscle volume. The volume of the muscular portion between every two consecutive scans was calculated with the following equation:

$$Volume = \frac{1}{3} * d * \left(a + \sqrt{(ab)} + b\right)$$

Where *a* and *b* are the anatomical cross-sectional areas of the muscle of two consecutive cross-sectional scans and *d* is the interval distance between the cross-sectional area measurements. The volume of the entire muscle was calculated by summing up all of the inter-scan muscular volumes [25]. Two full-length sagittal images were then recorded to allow for the measurement of resting BF_{LH} muscle fascicle length and pennation angle, which were both assessed in 3 fascicles at 50% of the total length of BF_{LH}. This point was measured offline (ImageJ). A comparison between offline (sagittal ultrasound) and tape measurements of the total BF_{LH} length revealed a very high correlation (R^2=0.96, P<0.001). Fascicle length was measured by tracing the fascicular path from the upper aponeurosis to the intra-muscular aponeurosis of the BF_{LH}. Muscle fascicle pennation angle was determined as the angle between the muscle fascicular paths and their insertion into the intra-muscular aponeurosis. The mean of the three measurements for each parameter were used to determine both fascicle length and pennation angle of the BF_{LH} muscle. PCSA was calculated by dividing BF_{LH} volume by its fascicle length. During the time of data collection, a similar methodological approach was published elsewhere [26]. One longitudinal image of one participant in the present study was not analysed due to low image quality. Ultrasound scans and image analysis was performed by the same investigator.

S11 Kinematic and Kinetic Data

Three-dimensional kinematic and kinetic data were synchronously collected at 500 Hz using an eight-camera motion analysis system (Qqus 300+; Qualisys, Gothenburg, Sweden). The data were filtered with a digital dual low-pass Butterworth filter at 20 Hz for motion, as previously described [27]. Retroreflective markers (12 mm diameter) were placed on anatomical landmarks on the right leg and pelvis, as previously described [28]. One standing static and two functional motion calibration trials were recorded of the participant PRE, POST and POST48. For the static trial, participants stood with their feet approximately shoulder width apart and knees fully extended. This static trial determined local coordinate systems, the location of joint centres, and the foot, shank, thigh, and pelvis segment lengths of each participant. The functional trials defined functional hip joint centres [29] and knee joint axes [30]. Kinematic data were tracked using Qualisys Track Manager Software (Qualisys). Data processing and

analysis were undertaken in Visual3D (C-Motion, Germantown, MD). To examine any changes between the time points, joint angles were normalised relative to the static trial of the accompanying time point for minimising the influence of potential slightly different marker positions between the trials. Lower extremity 3D joint angles and angular velocities were calculated using an X-Y-Z Cardan angle rotation sequence. Investigated variables included peak knee and hip angles, as well as range of motion and time during stance and swing phase of the treadmill run, for all three planes, were calculated as described in previous studies [27].

S12 Motorised Treadmill Run

Participants ran on a motorised treadmill (HP Cosmos Pulsar; Nussdorf, Germany) for 30 s at 4.17 m s^{-1} (0° incline), as high-speed running was of interest. The selected speed was based on pilot testing, which demonstrated that 15 km/h (4.17 m/s) was the fastest speed on a motorized treadmill where the participants still felt comfortable. Motion analysis data were recorded for the last 10 s of the run and data were analysed for at least 6 consecutive strides. Peak knee and hip angle data, for all three planes, were calculated (i) between the initial contact and terminal stance of foot; and (ii) between initial and terminal swing phase. The touchdown of the foot during the treadmill run was determined from the kinematic data as occurring at the local minima and the toe-off during running as the local maxima of the vertical velocity of the head of the fifth metatarsal marker on the foot [31].

S13 Blood Samples

A 10 mL blood sample was drawn from an antecubital vein in the forearm and collected into a serum vacutainer (BD Vacutainer systems, Plymouth, UK). The blood samples were obtained at each time point and left at 22-24°C for 30 min to allow clotting, and then kept on ice when necessary. Serum samples were centrifuged at 1,300 g for 15 min at 4°C. All samples were then aliquoted into 1.5 mL microcentrifuge tubes [Axygen (Corning), New York, USA] and stored at -80°C until subsequent analysis (see below).

S14 Serum Interleukin-6 (IL-6) Concentration

Serum samples were assayed for IL-6 concentration using commercially available human IL-6 enzyme linked immunosorbent assay kits (Quantikine®, R&D systems, Minneapolis, MN, USA) according to the manufacturer's instructions. The intensity of the colour produced after 20 min was measured with a Thermo Multiskan Spectrum microplate reader (Thermo Fisher Scientific. Waltham, MA. USA) at 450 nm and values were calculated with Excel 365 (Microsoft, v. 365, USA) by generating a four-parameter logistic curve fit. The minimum detectable dose of human IL-6 was 0.70 pg/mL.

S15 Serum Creatine Kinase Activity

Creatine kinase (CK) activity was assayed using a commercially available CK assay (Catachem Inc., Connecticut, NE, USA), as described in detail elsewhere [32]. Briefly, 10 µL blood serum were loaded onto a 96-well UV plate. The CK reaction reagent and diluent (Catachem) were prepared as per the manufacturer’s instructions and added to the samples and the change in absorbance monitored continuously over 20 min in a Thermo Multiskan Spectrum plate reader (Thermo Fisher Scientific. Waltham, MA. USA) at a wavelength of 340 nm.

S16 Capillary Blood Lactate Concentration

Capillary blood samples were taken from the finger-tip via a Safety-Lancet Extra 18G needle (Sarstedt; Nümbrecht, Germany) at rest before and immediately after the repeated maximal sprint intervention. Blood samples were analysed within 60 seconds of collection using a portable blood lactate analyser (Arkray Lactate Pro; Kyoto, Japan).

S17 Reagents, Chemicals, and Solvents for Muscle Cell Culture *in vitro*

Growth media used for the expansion of human muscle-derived cell populations consisted of Hams F-10 nutrient mix (Lonza, Basel, Switzerland) with added L-glutamine (2.5 mM), 10% heat-inactivated fetal bovine serum (hiFBS; Gibco, Thermo Fisher Scientific, Altincham, UK), 1% penicillin-streptomycin (Life Technologies, Warrington, UK), and 1% L-Glutamine (Gibco). Differentiation media consisted of α-MEM (Lonza), 1% hiFBS, 1% penicillin-streptomycin, and 1% L-glutamine. Phosphate-buffered saline (PBS; Sigma-Aldrich) was used to wash cell monolayers. Desmin polyclonal rabbit anti-human antibody (Cat# ab15200, RRID: AB_301744) was used (1:200) from Abcam (Abcam, Cambridge, UK), and secondary antibody (TRITC polyclonal goat anti-rabbit; Cat# A16101, RRID: AB_2534775) was used (1:200) from Fisher Scientific.

S18 Muscle Biopsy Procedure

Participants were instructed to avoid any strenuous exercise 48 h prior to the biopsy procedure. Biopsies from the vastus lateralis muscle were obtained under local anaesthesia from each participant, using the Weil-Blakesley conchotome technique as described previously [33]. The conchotome was inserted through the incision into the muscle belly to obtain the 134 ± 82.7 mg muscle biopsy.

S19 Extraction of Human Muscle-Derived Cells

The muscle biopsies analysed in this study were isolated and cultured [32], as reported previously. Briefly, biopsy samples were transferred with precooled growth media from the muscle biopsy suite to the sterile tissue culture hood (Kojair Biowizard Silverline class II hood; Kojair, Vippula, Finland) within 40 min and muscle biopsy samples were washed three times with ice-cold PBS (0.01 M phosphate buffer, 0.0027 M KCl, and 0.137 M NaCl, pH 7.4, in dH2O). Visible fibrous and fat tissue was removed using sterile scissor and forceps. Samples were cut in small pieces (1 mm^3) and digested in 5 ml of trypsin-EDTA for 15 min on a magnetic stirring platform at 37°C to dissociate muscle cells. The trypsinisation process was repeated twice. Supernatant derived following each treatment was collected and pooled with hiFBS at a concentration of 10% of the total volume to inhibit further protease activity. Cell supernatant was centrifuged at 450 g for 5 min. Supernatant was discarded and the cell pellet was resuspended in growth media and plated on a T25 cm^2 culture flask (Corning, Life Sciences, New York, USA) for cell population expansion. Culture flasks were previously coated with a 2 mg/l porcine gelatin solution (90–110 g, Bloom; Sigma-Aldrich, Dorset, UK) to support cell adhesion.

S20 Expansion of Extracted Cells

The medium was refreshed on the fourth day after the extraction procedure and subsequently every 48 h following two brief washes with PBS. Mononuclear cells were incubated in a HERAcell 150i CO_2 Incubator (Thermo Scientific, Cheshire, UK). T25 cm^2 culture flasks reached 80% confluence after aproximately 10 days and were passaged via trypsinisation. Cells were counted using Trypan Blue exclusion and re-plated on gelatinised T75 cm^2 culture flasks (Nunc, Roskilde, Denmark). The mononuclear cells were expanded until passage 3 and then frozen in GM with 10% dimethyl sulfoxide (DMSO) in liquid N_2 as a cryopreservant. All experiments were performed on cells between passages 3 and 6 to avoid potential issues of senescence [34].

S21 Characterization of Human Muscle-Derived Cells

Mononuclear cells, resident within the biopsies, were isolated and cultured *in vitro*, enabling determination of myoblast:fibroblast ratios. The mixed population of human skeletal muscle-derived mononuclear cells were characterised by immunofluorescent staining at passage 3 (about 7-10 days after the muscle stem cell isolation procedure before we froze the remaining cells down) for the detection of desmin expressed by myoblasts (desmin positive) and non-

myoblasts (desmin negative) to determine the percentage of myoblasts and fibroblasts. Grohmann, et al. [35] showed that passaging does not change the percentage of myoblast and fibroblasts and all populations were included for analysis. Previous investigations have determined that the non-myoblast (desmin negative) fraction is highly enriched in fibroblasts, with up to 99% of this fraction being fibroblasts [36,37], as our group also observed [32]. Therefore, non-myoblasts (desmin negative) were referred to here as fibroblasts.

Monolayers were incubated with 25% [vol/vol methanol in Tris-buffered saline (10 mmTris-HCl, pH 7.8, 150 mm NaCl)], 50% and 100% for 5-min to fix the cells and stored at 4°C wet in Tris-buffered saline until further analysis. Fixed monolayers were permeabilised and blocked for 2 h with 5% goat serum and 0.2% Triton X-100 in Tris-buffered saline, prior to staining. Cells were incubated overnight at 4°C with anti-Desmin antibody (1:200). After overnight incubation, the primary antibody was removed, and the cells were washed three times with Tris-buffered saline. Secondary TRITC polyclonal goat anti-rabbit antibody (1:200) was then applied and incubated for 2 h at 4°C. Fluorescent images were captured using live imaging microscopy (Leica DMB 6000; Magnification x 10.5) and analysed via ImageJ cell counter plug-in. A total of four randomly selected areas per well were analysed per individual.

S22 Wound-Healing Assay, Migration and Differentiation Analysis

For the wound healing assay, 100,000 cells/ml were seeded in gelatinised six-well plates (Nunc, Roskilde, Denmark). Cells were expanded as described above until cell monolayers reached a confluent state, Growth media was removed, monolayers were washed with PBS and cells were damaged by a vertical scrape with a 1-ml pipette tip (width of the wound area, *mean* ± *S.E.M.*: 896.4 ± 21.24 *µ*m), as previously reported by our group [32]. PBS was aspirated, damaged cell monolayers were washed twice with PBS to remove cell debris and 2 ml differentiation media was added. Monolayers were imaged with a live imaging microscopy (Leica) for the analysis of cell migration immediately, 24h, and 48h. TIF images were exported from Leica Application Suite and loaded as TIF image stacks in ImageJ with a cell counter plug-in. Cells in the outer and inner segments were then counted (Figure 3).

Damaged monolayers were imaged at two sites per well in the wound site immediately post-damage (0 h). These image coordinates were tracked and stored to allow subsequent monitoring of the same sites on the wound to reduce this experimental bias. Captured images were exported as TIF image files, and analysed in ImageJ.

S23 Statistical Analysis

One-way repeated-measures analysis of variances (ANOVA)s were performed to determine whether there was a significant main effect for time (within subject factor) for the following dependent variables: MVC torque, voluntary muscle activation, antagonist muscle co-activation, muscle soreness (for squat lunge via measured via visual analogue scale as well as algometer), rating of perceived exertion, CK activity, IL-6 concentration, and for kinematics data (hip and knee angle parameters). MVC torque data were analysed for interactions and main effects for muscle group and time using two-way mixed design ANOVAs, comparing differences between muscle groups across 3-time points; PRE, POST, and POST48. For within test comparisons, either, independent t-tests, or one-way ANOVAs were used where appropriate. For the torque-frequency relationship, normalised torque at each frequency was analysed using a two-way repeated measures ANOVA, with stimulation frequency (1-100 Hz) and time (PRE, POST and POST48) as the within-groups independent variables. Post-hoc one-way repeated measures ANOVAs were used to determine if the normalised torque at each frequency differed between time points. Bivariate correlations were used to analyse the relation between architectural parameters of the BF_{LH} (volume, fascicle length, fascicle pennation angle and PCSA) and fatigue biomarkers (relative MVC loss normalised to PRE MVC), serum CK activity, serum IL-6 concentration, muscle soreness, knee joint range of motion or changes in range of motion during treadmill running.

Bivariate correlations were used to analyse the relation between myoblast:fibroblast ratio and quadriceps and hamstring MVC, and migration dynamics (total cell migration, cell proportion of inner to outer segment) of the muscle stem cells. Standard guidelines concerning violation of the sphericity assumption to adjust the degree of freedom of the F-test by the Huynh-Felt epsilon if epsilon is greater than 0.75 and to use the more stringent Greenhouse-Geisser adjustment if epsilon is less than 0.75 were followed. Results were expressed as mean ± SD, unless otherwise stated, with statistical significance set at $P<0.05$. All MVC data were analysed with AcqKnowledge software 4.4 (Biopac-Systems Inc., Goleta, USA) and SPSS 23 Software (IBM Inc., Armonk, NY: IBM Corp) was used for statistical analysis. Occasional missing data are reflected in the reported degrees of freedom.

SUPPLEMENTARY TABLE

Table supplement 4 Architectural parameters of biceps femoris long head (mean ± SD).

**Muscle length [cm]**	**Fascicle length [cm]**	**PCSA [cm^2]**	**Volume [cm^3]**	**Fascicle Pennation Angle [°]**
27.86 ± 2.13	7.94 ± 1.38	23.4 ± 4.62	182.2 ± 29.5	12.7 ± 2.77

Fascicle length at 50% of BF_{LH} muscle length; PCSA – Physiological cross-sectional area.

REFERENCES

1 Wüst, R. C., Morse, C. I., De Haan, A., Jones, D. A. & Degens, H. Sex differences in contractile properties and fatigue resistance of human skeletal muscle. *Experimental physiology* **93**, 843-850 (2008).

2 Bradley, P. S., Di Mascio, M., Peart, D., Olsen, P. & Sheldon, B. High-intensity activity profiles of elite soccer players at different performance levels. *J. Strength Cond.* **24**, 2343-2351 (2010).

3 Girard, O., Mendez-Villanueva, A. & Bishop, D. Repeated-Sprint Ability—Part I. *Sports Med* **41**, 673-694 (2011).

4 Bradley, P. S. *et al.* High-intensity running in English FA Premier League soccer matches. *Journal of sports sciences* **27**, 159-168 (2009).

5 Timmins, R. G. *et al.* Reduced biceps femoris myoelectrical activity influences eccentric knee flexor weakness after repeat sprint running. *Scandinavian journal of medicine & science in sports* **24**, e299-e305 (2014).

6 Chen, C.-H., Ye, X., Wang, Y.-T., Chen, Y.-S. & Tseng, W.-C. Differential Effects of Different Warm-up Protocols on Repeated Sprints-Induced Muscle Damage. *J. Strength Cond.* **32**, 3276-3284 (2018).

7 Verma, S., Moiz, J. A., Shareef, M. Y. & Husain, M. E. Physical performance and markers of muscle damage following sport-specific sprints in male collegiate soccer players: repeated bout effect. *The Journal of sports medicine and physical fitness* **56**, 765-774 (2015).

8 Howatson, G. & Milak, A. Exercise-induced muscle damage following a bout of sport specific repeated sprints. *J. Strength Cond.* **23**, 2419-2424 (2009).

9 Glaister, M., Howatson, G., Pattison, J. R. & McInnes, G. The reliability and validity of fatigue measures during multiple-sprint work: an issue revisited. *J. Strength Cond.* **22**, 1597-1601 (2008).

10 van Dyk, N. *et al.* Hamstring and Quadriceps Isokinetic Strength Deficits Are Weak Risk Factors for Hamstring Strain Injuries A 4-Year Cohort Study. *The American journal of sports medicine* **44**, 1789-1795 (2016).

11 Guex, K., Gojanovic, B. & Millet, G. P. Influence of hip-flexion angle on hamstrings isokinetic activity in sprinters. *Journal of athletic training* **47**, 390-395, doi:10.4085/1062-6050-47.4.04 (2012).

12 Erskine, R. M., Jones, D. A., Maganaris, C. N. & Degens, H. In vivo specific tension of the human quadriceps femoris muscle. *Eur. J. Appl. Physiol.* **106**, 827-838 (2009).
13 Nedelec, M. *et al.* The influence of soccer playing actions on the recovery kinetics after a soccer match. *J. Strength Cond.* **28**, 1517-1523 (2014).
14 Kirk, E. A. & Rice, C. L. Contractile function and motor unit firing rates of the human hamstrings. *J. Neurophysiol.* **117**, 243-250 (2017).
15 Thelen, D. G. *et al.* Hamstring muscle kinematics during treadmill sprinting. *Medicine and science in sports and exercise* **37**, 108-114 (2005).
16 Erskine, R. M., Jones, D. A., Williams, A. G., Stewart, C. E. & Degens, H. Resistance training increases in vivo quadriceps femoris muscle specific tension in young men. *Acta physiologica* **199**, 83-89 (2010).
17 Marshall, P. W., Lovell, R., Jeppesen, G. K., Andersen, K. & Siegler, J. C. Hamstring muscle fatigue and central motor output during a simulated soccer match. *PLoS One* **10**, e102753 (2014).
18 Reeves, N. D., Narici, M. V. & Maganaris, C. N. In vivo human muscle structure and function: adaptations to resistance training in old age. *Experimental physiology* **89**, 675-689 (2004).
19 Kellis, E. & Baltzopoulos, V. In vivo determination of the patella tendon and hamstrings moment arms in adult males using videofluoroscopy during submaximal knee extension and flexion. *Clinical Biomechanics* **14**, 118-124 (1999).
20 Hermens, H. J., Freriks, B., Disselhorst-Klug, C. & Rau, G. Development of recommendations for SEMG sensors and sensor placement procedures. *Journal of electromyography and Kinesiology* **10**, 361-374 (2000).
21 Araujo, R. C., Duarte, M. & Amadio, A. C. On the inter-and intra-subject variability of the electromyographic signal in isometric contractions. *Electromyogr. Clin. Neurophysiol.* **40**, 225-230 (2000).
22 Lanza, M. B., Balshaw, T. G., Massey, G. J. & Folland, J. P. Does normalization of voluntary EMG amplitude to MMAX account for the influence of electrode location and adiposity? *Scand J Med Sci Sports* **28**, 2558-2566, doi:10.1111/sms.13270 (2018).
23 Scott, J. & Huskisson, E. Vertical or horizontal visual analogue scales. *Ann. Rheum. Dis.* **38**, 560 (1979).
24 Ekstrand, J., Hägglund, M. & Waldén, M. Epidemiology of muscle injuries in professional football (soccer). *The American journal of sports medicine* **39**, 1226-1232 (2011).
25 Erskine, R. *et al.* The individual and combined effects of obesity-and ageing-induced systemic inflammation on human skeletal muscle properties. *Int. J. Obes. (Lond.)* **41**, 102-111 (2017).
26 Seymore, K. D., Domire, Z. J., DeVita, P., Rider, P. M. & Kulas, A. S. The effect of Nordic hamstring strength training on muscle architecture, stiffness, and strength. *Eur. J. Appl. Physiol.* **117**, 943-953 (2017).
27 Verheul, J., Clansey, A. C. & Lake, M. J. Adjustments with running speed reveal neuromuscular adaptations during landing associated with high mileage running training. *Journal of applied physiology* **122**, 653-665, doi:10.1152/japplphysiol.00801.2016 (2017).
28 McClay, I. & Manal, K. Three-dimensional kinetic analysis of running: significance of secondary planes of motion. *Medicine and Science in Sports and Exercise* **31**, 1629-1637 (1999).
29 Schwartz, M. H. & Rozumalski, A. A new method for estimating joint parameters from motion data. *Journal of biomechanics* **38**, 107-116 (2005).
30 Robinson, M. A. & Vanrenterghem, J. An evaluation of anatomical and functional knee axis definition in the context of side-cutting. *Journal of biomechanics* **45**, 1941-1946 (2012).
31 Maiwald, C., Sterzing, T., Mayer, T. & Milani, T. Detecting foot-to-ground contact from kinematic data in running. *Footwear Science* **1**, 111-118 (2009).
32 Owens, D. J. *et al.* A systems-based investigation into vitamin D and skeletal muscle repair, regeneration, and hypertrophy. *Am. J. Physiol.-Endoc. M.* **309**, E1019-E1031 (2015).
33 Baczynska, A. M. *et al.* Human Vastus Lateralis Skeletal Muscle Biopsy Using the Weil-Blakesley Conchotome. *Journal of Visualized Experiments*, e53075-e53075 (2016).

34 Foulstone, E. J., Huser, C., Crown, A. L., Holly, J. M. & Stewart, C. E. Differential signalling mechanisms predisposing primary human skeletal muscle cells to altered proliferation and differentiation: roles of IGF-I and TNFα. *Experimental cell research* **294**, 223-235 (2004).

35 Grohmann, M. *et al.* Isolation and validation of human prepubertal skeletal muscle cells: maturation and metabolic effects of IGF-I, IGFBP-3 and TNFα. *J. Physiol.* **568**, 229-242 (2005).

36 Agley, C. C., Rowlerson, A. M., Velloso, C. P., Lazarus, N. R. & Harridge, S. D. Human skeletal muscle fibroblasts, but not myogenic cells, readily undergo adipogenic differentiation. *Journal of cell science* **126**, 5610-5625 (2013).

37 Stewart, J. D. *et al.* Characterization of proliferating human skeletal muscle-derived cells in vitro: Differential modulation of myoblast markers by TGF-β2. *J. Cell. Physio.* **196**, 70-78 (2003).
